# Unlocking nitrogen management potential via large-scale farming for air quality and substantial co-benefits

**DOI:** 10.1093/nsr/nwae324

**Published:** 2024-09-13

**Authors:** Baojie Li, Hong Liao, Ke Li, Ye Wang, Lin Zhang, Yixin Guo, Lei Liu, Jingyi Li, Jianbing Jin, Yang Yang, Cheng Gong, Teng Wang, Weishou Shen, Pinya Wang, Ruijun Dang, Kaihua Liao, Qing Zhu, Daniel J Jacob

**Affiliations:** Collaborative Innovation Center of Atmospheric Environment and Equipment Technology, Jiangsu Key Laboratory of Atmospheric Environment Monitoring and Pollution Control, School of Environmental Science and Engineering, Nanjing University of Information Science and Technology, Nanjing 210044, China; Collaborative Innovation Center of Atmospheric Environment and Equipment Technology, Jiangsu Key Laboratory of Atmospheric Environment Monitoring and Pollution Control, School of Environmental Science and Engineering, Nanjing University of Information Science and Technology, Nanjing 210044, China; Collaborative Innovation Center of Atmospheric Environment and Equipment Technology, Jiangsu Key Laboratory of Atmospheric Environment Monitoring and Pollution Control, School of Environmental Science and Engineering, Nanjing University of Information Science and Technology, Nanjing 210044, China; Collaborative Innovation Center of Atmospheric Environment and Equipment Technology, Jiangsu Key Laboratory of Atmospheric Environment Monitoring and Pollution Control, School of Environmental Science and Engineering, Nanjing University of Information Science and Technology, Nanjing 210044, China; Laboratory for Climate and Ocean-Atmosphere Studies, Department of Atmospheric and Oceanic Sciences, School of Physics, Peking University, Beijing 100871, China; Earth, Ocean and Atmospheric Sciences Thrust, Function Hub, Hong Kong University of Science and Technology (Guangzhou), Guangzhou 511442, China; College of Earth and Environmental Sciences, Lanzhou University, Lanzhou 730000, China; Collaborative Innovation Center of Atmospheric Environment and Equipment Technology, Jiangsu Key Laboratory of Atmospheric Environment Monitoring and Pollution Control, School of Environmental Science and Engineering, Nanjing University of Information Science and Technology, Nanjing 210044, China; Collaborative Innovation Center of Atmospheric Environment and Equipment Technology, Jiangsu Key Laboratory of Atmospheric Environment Monitoring and Pollution Control, School of Environmental Science and Engineering, Nanjing University of Information Science and Technology, Nanjing 210044, China; Collaborative Innovation Center of Atmospheric Environment and Equipment Technology, Jiangsu Key Laboratory of Atmospheric Environment Monitoring and Pollution Control, School of Environmental Science and Engineering, Nanjing University of Information Science and Technology, Nanjing 210044, China; Department of Biogeochemical Signals, Max Planck Institute for Biogeochemistry, Jena 07745, Germany; College of Oceanography, Hohai University, Nanjing 210024, China; Collaborative Innovation Center of Atmospheric Environment and Equipment Technology, Jiangsu Key Laboratory of Atmospheric Environment Monitoring and Pollution Control, School of Environmental Science and Engineering, Nanjing University of Information Science and Technology, Nanjing 210044, China; Collaborative Innovation Center of Atmospheric Environment and Equipment Technology, Jiangsu Key Laboratory of Atmospheric Environment Monitoring and Pollution Control, School of Environmental Science and Engineering, Nanjing University of Information Science and Technology, Nanjing 210044, China; John A. Paulson School of Engineering and Applied Sciences, Harvard University, Cambridge, MA 02138, USA; Key Laboratory of Watershed Geographic Sciences, Nanjing Institute of Geography and Limnology, Chinese Academy of Sciences, Nanjing 210008, China; Key Laboratory of Watershed Geographic Sciences, Nanjing Institute of Geography and Limnology, Chinese Academy of Sciences, Nanjing 210008, China; John A. Paulson School of Engineering and Applied Sciences, Harvard University, Cambridge, MA 02138, USA

**Keywords:** nitrogen management, large-scale farming, air quality, ammonia emission

## Abstract

China's sustained air quality improvement is hindered by unregulated ammonia (NH_3_) emissions from inefficient nitrogen management in smallholder farming. Although the Chinese government is promoting a policy shift to large-scale farming, the benefits of this, when integrated with nitrogen management, remain unclear. Here we fill this gap using an integrated assessment, by combining geostatistical analysis, high-resolution emission inventories, farm surveys and air quality modeling. Smallholder-dominated farming allows only 13%–31% NH_3_ reduction, leading to limited PM_2.5_ decreases nationally due to non-linear PM_2.5_ chemistry. Conversely, large-scale farming would double nitrogen management adoption rates, increasing NH_3_ reduction potential to 48%–58% and decreasing PM_2.5_ by 9.4–14.0 μg·m^−3^ in polluted regions. The estimated PM_2.5_ reduction is conservative due to localized NH_3_-rich conditions under large-scale livestock farming. This strategy could prevent over 300 000 premature deaths and achieve a net benefit of US $68.4–86.8 billion annually, unlocking immense benefits for air quality and agricultural sustainability.

## INTRODUCTION

Agricultural ammonia (NH_3_) emissions are making increasing contributions to global air pollution, given the effective control of acidic gas emissions [1]. As a major alkaline gas, NH_3_ facilitates the formation of secondary inorganic aerosols, such as ammonium sulfate/bisulfate and ammonium nitrate, thus contributing to PM_2.5_ (fine particles with aerodynamic diameters of <2.5 μm). However, in China, NH_3_ remains the sole unregulated major atmospheric pollutant, leading to the most rapidly growing national NH_3_ level globally [2,3]. China's 14th Five-Year Plan proposes only a 5% reduction in NH_3_ emissions from large-scale livestock farms in heavily polluted regions—a target that offers extremely limited potential for achieving substantial air quality improvements [4]. Hence, there is an urgent need to establish an efficient NH_3_ reduction strategy in China to improve air quality and promote sustainable agricultural development.

Fertilizer application and livestock waste account for >80% of NH_3_ emissions, both in China [5,6] and globally [7,8]. Numerous studies have explored NH_3_ reduction strategies, including technological advancements (e.g. reduced overuse of N fertilizer and advanced manure management) [9–11], adjusting trade structures by increasing imports from nations with lower NH_3_ emission intensity [12,13], and optimizing human diets with less animal-derived products [14,15]. However, substantial regional variations in diet and culture pose considerable constraints on the proposed strategies. Currently, the most efficient and feasible approach is nitrogen (N) management relying on advanced technology in China. For example, the use of enhanced-efficiency N fertilizers can reduce NH_3_ emissions by >40% for all crops [16].

However, mobilizing >200 million smallholder farmers to adopt such advanced technologies is unfeasible under the current traditional farming regime. Large numbers of older farmers tend to adhere to long-standing agricultural practices (e.g. overuse of N fertilizer), while the younger generation has little incentive to change these practices as they allocate more time to higher-earning non-agricultural work [17]. The purported NH_3_ mitigation potential of technological advancements (e.g. 50% reduction [11] and 38%–67% reduction [14]) may be overly optimistic, assuming 100% adoption rates that are unrealistic for smallholder farms given practical constraints. Agricultural regime transformations are necessary for further NH_3_ decreases.

Implementation of large-scale farming, characterized by expanded cropland farm sizes per parcel through land consolidation and by the transition from free-range to large-scale animal husbandry, has been explicitly and repeatedly promoted by the Chinese government (Table S1 in Supplementary Data online) as a key approach to promoting agricultural productivity and mitigating agricultural pollution [4,18]. There has been a substantial expansion of large (>6.7 ha) crop farms (8.9% annual growth) and large livestock farms (6.6% annual growth) in recent years (Fig. S1 in Supplementary Data online). Larger farm sizes and more livestock enable more knowledge exchange and mechanization, reducing barriers for farmers to embrace advanced N management techniques (e.g. precision N fertilizer application) [19]. However, understanding the potential benefit of integrating N management and large-scale farming on NH_3_ emissions and air quality remains limited.

Here, we aim to quantify the reductions in NH_3_ emissions and associated effects on air quality and economic benefits under current and proposed large-scale farming regimes in China. We predict the distributions of farm size and livestock production under a large-scale farming regime based on cropland spatial connectivity, geostatistical analysis and real-world geographic coordinates of livestock farms. Five NH_3_ abatement scenarios (three under the current regime, and two under a large-scale farming regime, as detailed in Methods) are developed using high-resolution emissions inventories along with several N management practices and advanced technology adoption rates from surveys of >18 000 farms. Air quality and related health improvements are assessed using the Goddard Earth Observing System Chemical Transport Model (GEOS-Chem) and Global Exposure Mortality Model (GEMM). Finally, the costs, private benefits and societal benefits of integrating large-scale farming and N management are evaluated via cost–benefit analysis across a range of economic parameters.

## RESULTS

### Quantification of large-scale farming

Large-scale farming is usually defined as farm size per parcel >2 ha for crop farms or the number of livestock per farm exceeding certain thresholds (e.g. >500 pigs, >100 dairy cattle) in China (Table S2), a definition widely adopted by the scientific community and policy makers [20–22]. Large-scale farming features expanded cropland farm sizes per parcel and transitions from free-range to large-scale animal husbandry, alongside increased adoption of advanced N management technologies. We predicted the farm size distribution achievable under a large-scale farming strategy by considering not only cropland spatial connectivity, but also terrain slopes using high-resolution digital elevation model (DEM) data, a factor overlooked in previous studies [18]. We found that 22.7% of China's cropland possesses slopes exceeding 6°, which may hinder cost-effective large-scale farming (or mechanization). Additionally, we obtained real-world geographic coordinates of large-scale livestock farms using points of interest (POI) data from Amap (https://lbs.amap.com/) to improve the allocation accuracy of current livestock production. The spatial reallocation of livestock production after large-scale farming was achieved by allocating free-range animals to large-scale livestock farms within respective provinces. Data on advanced technology adoption were obtained from an extensive literature review of 18 967 farm surveys (see Methods).

**Figure 1.**
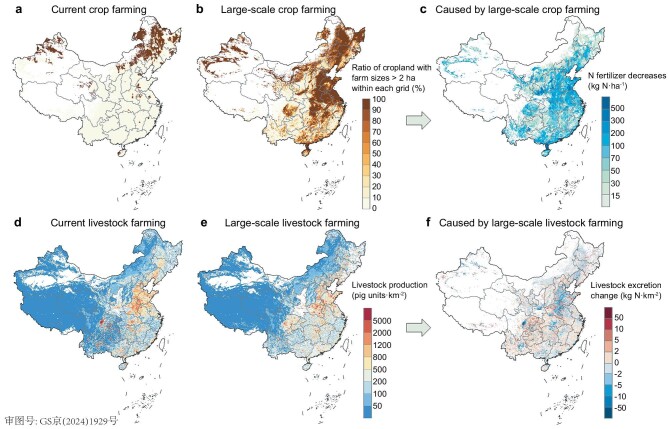
Quantification of large-scale crop and livestock farming. The increased proportion of croplands achieving large-scale farming (farm size > 2 ha) within each 5’ × 5’ grid from (a) the current regime to (b) the large-scale crop farming regime, results in a (c) decrease in N fertilizer rates. The transition of livestock production from (d) the current regime to (e) large-scale farming redistributes (f) manure N emissions. Livestock production was converted to pig units, where one head of dairy cattle, beef cattle, sheep/goats, layer poultry and broiler poultry is equivalent to 10, 5, 1/3, 1/15 and 1/60 pig units, respectively. Data from Hong Kong, Macao and Taiwan are not available in this study.

China had 134.9 million ha of cropland in 2016, of which 80.8% was in parcels of <2 ha, primarily managed by smallholders. Following land consolidation, while the total arable land area would remain unchanged, the fraction of large-scale crop farms would increase by 300.9% (from 25.9 to 103.6 million ha), achieved by merging fragmented parcels into larger units. Figure 1 shows the fractions of large-scale crop farming (farm size > 2 ha) within each 5’ × 5’ grid cell (∼8400 ha) under the current and large-scale regimes. Based on field surveys, farm size is a strong factor affecting fertilizer use per hectare in China [23]. We calculated the potential for decreased N fertilizer application rates from large-scale farming based on farm size changes and current gridded N fertilizer application rates (see Methods). Currently, the mean N application rate in China is 197.8 kg N·ha^−1^. With large-scale crop farming, this would reduce by 61.5 kg N·ha^−1^ (31.07%), with the fastest declines in the North China Plain, Middle–Lower Yangtze Plain and Sichuan Basin (SCB) (Fig. 1).

In the livestock sector, the total national production was 1.6 billion pig units in 2016, converted from heads of swine, cattle, sheep and poultry. Among these, 1.0 billion pig units were from smallholder free-range farming, contributing 7.2 Tg N in manure N emission. Such free-range livestock excretions led to non-point source NH_3_ pollution and generally poor manure management practices. With large-scale livestock farming, the production in large-scale livestock farms would surge to 92.8% (1.5 billion pig units) of total national production, with major spatial redistribution of manure N emissions, which would subsequently influence NH_3_ emissions. The largest decreases in N application rates and manure N emissions often overlap with hotspots of PM_2.5_ pollution, pointing to the effectiveness of large-scale farming in mitigating air pollution.

Additionally, the implementation of large-scale farming can significantly improve the adoption rates of advanced N management technologies. Based on 18 967 farm surveys (see Methods), we found that the adoption rates of advanced technologies are 22.2% for smallholder crop farms, 43.8% for large-scale crop farms, 19.6% for smallholder livestock farms and 39.1% for large-scale livestock farms in China (Fig. S2). Importantly, the doubling of adoption rates directly enhances the efficacy of emissions mitigation strategies as well as achievable NH_3_ reductions.

### Potential reductions of NH_3_ emissions under smallholder and large-scale farming regimes

**Figure 2.**
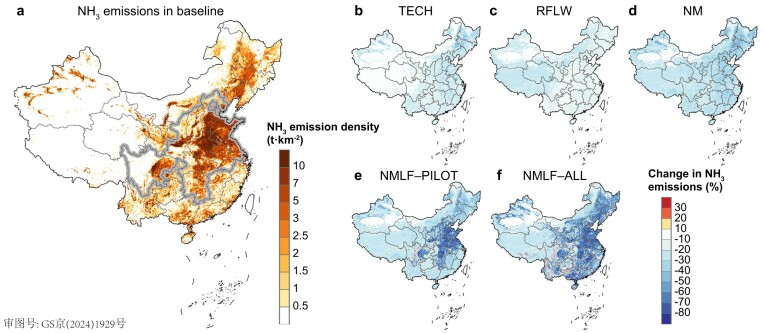
NH_3_ emissions and potential reductions under current and large-scale farming regimes. (a) Baseline NH_3_ emissions in 2016. Percentage reductions (%) in NH_3_ emissions under different scenarios: (b) using advanced technology solely (TECH), (c) reduced food loss and waste (RFLW), (d) N management under the current farming regime (NM, a combination of TECH and RFLW), (e) N management nationwide following large-scale farming in pilot areas (NMLF–PILOT) and (f) N management after implementing large-scale farming throughout China (NMLF–ALL). Pilot regions are shown within gray boundaries in (a). Data from Hong Kong, Macao and Taiwan are not available in this study.

Annual NH_3_ emissions in China were calculated to be 12.8 Tg (11.1–16.0 Tg, 90% confidence intervals, CI, based on Monte Carlo simulation) in 2016 (Fig. 2), which is within the range obtained by other studies [6,11,24,25] (Text S1 in Supplementary Data online). Livestock waste (5.9 Tg; CIs, 4.4–7.2 Tg) and fertilizer application (4.9 Tg; CIs, 4.1–7.6 Tg) accounted for 46.1% and 38.2% of the total, respectively (see Table S3 for specific sources). NH_3_ emissions were highest in summer (4.7 Tg) and lowest in winter (1.8 Tg) (Fig. S3).

Five NH_3_ emission reduction scenarios were developed. Among them, three were designed to assess NH_3_ mitigation potential under the current smallholder-dominated farming regime: (i) using advanced technology solely (TECH, seven abatement technologies considered), (ii) reduced food loss and waste (RFLW) and (iii) N management (NM, a combination of TECH and RFLW). Two additional scenarios were developed for large-scale farming: (iv) N management throughout China following the implementation of large-scale farming in pilot areas (10 provinces with high concentrations of PM_2.5_ and high NH_3_ emissions, which account for only 20.8% of China's total land area, see Fig. 2a; NMLF–PILOT) and (v) N management after implementing large-scale farming throughout China (NMLF–ALL). For the different abatement scenarios, areas with large reductions in NH_3_ emissions were found to be concentrated in hotspots of NH_3_ emissions, such as the North China Plain (Fig. 2).

Under the current regime, the mitigation potential when considering only technological advancement (TECH scenario) would reach 21.2% in China (Table 1). This emission reduction value is notably lower than that of other studies (33% [10], 50% [11] and 38%–67% [14]), which may be related to the overly optimistic adoption ratios of N management technologies used in those studies. As a consumption-side strategy, the RFLW scenario leads to a 12.7% decrease in emissions, which is achieved by reducing supply-chain food loss and encouraging people (particularly urban residents) to reduce their food loss and waste [26]. Implementing N management under the current traditional farming regime (NM) is estimated to reduce total NH_3_ emissions in China by 30.9%. Even in Beijing–Tianjin–Hebei and its surrounding areas (BTH, see Fig. S4), which have a high NH_3_ emission density, the emission reduction is only 32.7%.

**Table 1.** NH_3_ emissions under the baseline and different abating strategies for current and large-scale farming regimes in China.

			Subregions			
		China^a^	BTH^g^	YRD^h^	SCB^i^	PILOT^j^
**NH_3_ emissions (Tg)**						
Baseline^b^	Total	12.8	1.9	1.2	0.9	7.0
	Fertilizer application	4.9	0.8	0.6	0.3	3.1
	Livestock waste	5.9	0.8	0.4	0.4	2.8
**NH_3_ reductions (%)**						
TECH^c^	Combined	21.2	23.2	21.0	16.6	20.9
(1) Use of enhanced-efficiency fertilizers		5.3	4.6	6.5	4.2	5.0
(2) Reduce overuse of N fertilizer		2.7	3.1	3.0	2.2	2.9
(3) Deep fertilizer placement		4.4	5.7	7.1	2.5	5.3
(4) Low crude protein feeding		2.2	2.8	1.9	1.9	2.3
(5) Manure management		9.7	10.7	6.7	8.1	8.9
RFLW^d^	Combined	12.7	13.0	9.9	11.7	12.1
(1) Reduce meat loss and waste		10.6	10.8	7.2	9.7	9.6
(2) Reduce staple food loss and waste		0.8	0.8	1.1	0.6	0.8
(3) Reduce loss and waste of fruits and vegetables		1.3	1.5	1.6	1.4	1.6
NM^e^		30.9	32.7	28.4	25.9	30.0
NMLF–PILOT^f^		48.3	63.3	58.6	52.1	61.9
NMLF–ALL^f^		58.2	64.4	61.8	58.8	61.9

^a^Annual NH_3_ emission levels are provided for all of China and for four subregions. ^b^Baseline refers to 2016 values. ^c^TECH scenario represents only the implementation of technological advancements. ^d^RFLW represents a reduction in food loss and waste. ^e^NM refers to agricultural nitrogen management under traditional farming regimes (combination of TECH and RFLW). ^f^NMLF–PILOT and NMLF–ALL are scenarios for implementing nitrogen management nationwide after large-scale farming in the pilot region and across all regions of China, respectively. ^g^BTH is a 2 + 26 (Beijing, Tianjin and 26 other municipalities in the surrounding area) region. ^h^YRD, Yangtze River Delta. ^i^SCB, Sichuan Basin (Fig. S4). ^j^PILOT represents the 10 provinces with high concentrations of PM_2.5_ as well as high NH_3_ emissions, as shown by the gray boundary in Fig. 2a.

Implementing N management nationwide following large-scale farming in pilot areas (NMLF–PILOT) would reduce total NH_3_ emissions by 48.3% throughout China, with a reduction potential of 61.9% within the pilot areas. In the BTH region, the reduction would reach 63.3%. If large-scale farming was implemented throughout the whole of China (NMLF–ALL), the reduction efficiency would reach 58.2%, with fertilizer application and livestock waste contributing 27.5% and 30.7%, respectively. Among different regions, fertilizer application in the Yangtze River Delta (YRD) contributed the most (41.1%) to its total NH_3_ reduction, while livestock waste in the SCB played the dominant role (34.0%) in reducing NH_3_ emissions.

### Impacts of abating strategies on air quality and health burden

**Figure 3.**
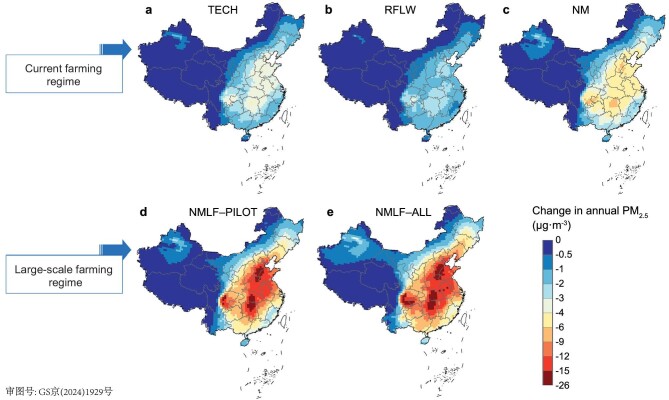
Air quality improvements following implementation of NH_3_ abatement strategies under two agricultural regimes. Changes in annual mean ground-level PM_2.5_ concentrations under the current smallholder-dominated farming regime with implementation of the (a) TECH, (b) RFLW, (c) NM strategies; and those under the large-scale farming regime with implementation of the (d) NMLF–PILOT and (e) NMLF–ALL strategies. All results are relative to the baseline simulation calculated via the GEOS-Chem model (0.625° × 0.5° horizontal resolution). Data from Hong Kong, Macao and Taiwan are not available in this study.

The GEOS-Chem model was employed to simulate the impact of NH_3_ abatement strategies on PM_2.5_ concentrations. Under the current smallholder-dominated and large-scale farming regimes, decreases in mean annual PM_2.5_ concentrations across China range from 4.2% to 10.0% and from 19.2% to 22.8%, respectively (Fig. 3; Table S4). Improving advanced technology alone (TECH scenario) would reduce the mean PM_2.5_ in China by 1.0 μg·m^−3^ (6.2%); the largest NH_3_ reduction (30.9%) under the current farming regime (NM scenario) had a simulated annual mean reduction of 1.6 μg·m^−3^, with declines in the major polluted regions (including the pilot region, BTH, YRD and SCB) reaching only 3.9–5.4 μg·m^−3^ (Table S4). The limited potential of reducing NH_3_ emissions under the current traditional farming regime is compounded by the non-linear response of PM_2.5_ to ammonia reduction. Small decreases in ammonia emissions have little benefit for decreasing PM_2.5_ under ammonia-rich conditions [27].

Under the NMLF–PILOT scenario, the reduction in national annual PM_2.5_ concentration would be 3.1 and 1.9 times that of the TECH scenario and NM scenario, respectively, primarily enabled by substantially increased advanced technology adoption under large-scale farming. In China's most severely polluted BTH region, the annual average PM_2.5_ concentration decrease could reach 13.4 μg·m^−3^ (up to 24.7 μg·m^−3^) under the NMLF–PILOT scenario, which is 2.5 times higher than the reductions obtained under the NM scenario (Table S4). Seasonally, NH_3_ emission reductions would be 3.3 times larger in summer than in winter, yielding higher reduction in PM_2.5_ levels in summer (25.6%) than in winter (15.5%). The largest reductions in PM_2.5_ would be seen for Central China and the SCB in winter and for the North China Plain in summer (Fig. S5).

Moreover, PM_2.5_ concentrations under the NMLF–ALL scenario would decrease only slightly compared with those under NMLF–PILOT, especially in heavily polluted areas (Table S4). Since the pilot region contains most of the PM_2.5_ and NH_3_ emission hotspots in China, additional emission reductions outside the pilot region under NMLF–ALL would have a limited effect (3.5%, 0.57 μg·m^−3^).

**Figure 4.**
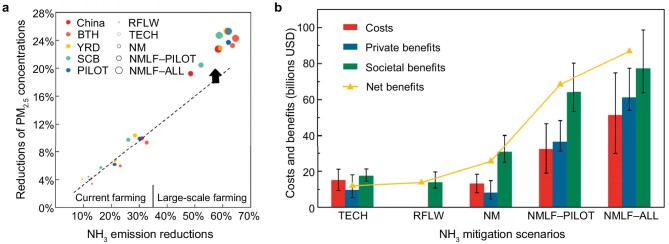
NH_3_ reductions for PM_2.5_ control and cost–benefit analysis under different abatement strategies. (a) Non-linear response of PM_2.5_ decline (%) to NH_3_ reduction rate (%) under the RFLW, TECH, NM, NMLF–PILOT and NMLF–ALL scenarios. (b) Cost–benefit analysis of the different strategies. Total costs, private benefits, societal benefits and net benefits of different scenarios are shown using mid-level economic parameters, with error bars representing low- and high-level estimates.

PM_2.5_ control efficiency (defined as the percentage reduction of PM_2.5_ divided by the percentage reduction of NH_3_ emission) in China under the NMLF–PILOT scenario (0.40%/%) would surpass the efficiency observed under the current traditional farming regime (0.29%/%–0.32%/%) and slightly exceed that of the NMLF–ALL scenario (0.39%/%) (Fig. 4a). Consequently, the targeted NH_3_ emissions reduction strategy of the NMLF–PILOT scenario emerges as the most efficient for controlling PM_2.5_; thus, implementing large-scale farming and N management in this pilot region is an ideal option for the near future. Among regions, SCB exhibits the highest PM_2.5_ control efficiency (e.g. 0.42%/% vs. the national average of 0.39%/% under the NMLF–ALL scenario), primarily due to its unfavorable dispersion conditions that lead to a stronger response of PM_2.5_ to NH_3_ reductions. Additionally, NH_3_ reductions achieved under the NMLF–PILOT and NMLF–ALL scenarios would both result in significant reductions in the frequency of heavily polluted days (daily mean PM_2.5_ > 150 μg·m^−3^) by 60.9% and 63.5%, respectively, which are substantially higher than those achieved through NH_3_ reductions under the current traditional farming regime (17.2%–33.9%; Fig. S6). Thus, integrating large-scale farming and N management can significantly reduce peak PM_2.5_ concentrations, thereby more effectively mitigating air pollution.

Notably, the PM_2.5_ reductions simulated by GEOS-Chem for large-scale farming are likely conservative. This is because transitioning livestock production from diffuse to concentrated point sources can lead to strongly localized NH_3_-rich conditions. A large fraction of NH_3_ in these concentrated plumes may deposit to the surface before diluting to the regional scale where PM_2.5_ formation becomes NH_3_-sensitive [28,29]. This effect cannot be captured by our GEOS-Chem simulation at ∼50 km resolution. We applied a 5-km-resolution box model to simulate the impacts of heterogeneous NH_3_ emission distribution within one GEOS-Chem grid cell (see Methods). The underestimation of the reduction in average PM_2.5_ concentration in a GEOS-Chem grid cell increases with the enhancement of the localized NH_3_-rich environment, reaching a maximum of 11.3% in the grid cell average PM_2.5_ concentration when livestock NH_3_ emissions are concentrated into a single 25 km^2^ box (Fig. S7).

The decrease in PM_2.5_ afforded by different NH_3_ mitigation strategies can be translated into a potential decrease in premature mortality (Fig. S8, Table S5). Under the current traditional farming regime, the maximum NH_3_ emissions reduction could avoid 120 000 PM_2.5_-related premature deaths in China annually; whereas TECH and RFLW could reduce deaths by 74 300 and 48 600 annually, respectively. Following the implementation of large-scale farming, the number of avoided premature deaths under NMLF–PILOT (256 000) is more than double that under NM, equating to 14.3% of all 1.79 million premature deaths attributed to PM_2.5_ exposure in China. This number increases to 302 000 people under NMLF–ALL.

### Cost–benefit analysis for abating NH_3_ emissions

Consideration of agricultural policies must consider the ensemble of correlated costs, private benefits (e.g. labor saving) and societal benefits (e.g. benefits to human health by reducing PM_2.5_, ecosystem health and greenhouse gas (GHG) mitigation) [10,30] (detailed in Methods). Table 2 lists the specific costs, private benefits and societal benefits of different NH_3_-emission-abatement scenarios using cost–benefit analysis. All five scenarios are estimated to have positive net benefits per annum (Fig. 4b), equating to US$11.9 billion (TECH), 13.8 billion (RFLW), 25.6 billion (NM), 68.4 billion (NMLF–PILOT) and 86.8 billion (NMLF–ALL). Notably, the NMLF scenarios provide net benefit at a private level, meaning that they pay for themselves. The NM scenarios provide net benefit only when considering the societal component. Among the proposed strategies, NMLF–PILOT possesses the highest benefit–cost ratio at 3.13, demonstrating the most favorable cost-effectiveness.

**Table 2.** Costs and benefits for different NH_3_ mitigation scenarios in US$ billions per annum.

**Cost and benefits**	TECH	RFLW	NM	NMLF-PILOT	NMLF-ALL
**Total costs**	**15.0^a^**	**/**	**13.0**	**32.1**	**51.3**
Using enhanced-efficiency fertilizers	4.2	/	4.1	4.0	3.6
Deep fertilizer placement	3.6	/	3.5	0.5	0.6
Manure management	7.1	/	5.5	4.2	3.8
Large-scale crop farming^b^	/	/	/	11.3	19.4
Large-scale livestock farming^c^	/	/	/	12.1	24.0
**Total benefits**	**26.9**	**13.8**	**38.6**	**100.5**	**138.1**
**Total private benefits**	**9.5**	/	**8.0**	**36.5**	**61.0**
Reducing overuse of N fertilizer	1.1	/	1.1	4.8	6.6
Deep fertilizer placement	2.5		2.4	3.2	3.8
Low crude protein feeding	2.2	/	1.6	2.1	2.4
Manure management^d^	3.7	/	2.8	4.7	6.4
Benefits from large-scale crop farming except for the reduction of chemical fertilizer^e^	/	/	/	8.5	16.0
Profits expansion after large-scale livestock farming^f^	/	/	/	13.3	25.7
**Total societal benefits**	**17.4**	**13.8**	**30.7**	**64.1**	**77.1**
Greenhouse gas mitigation benefit	0.8	2.8	3.5	4.6	7.2
Human health benefit	18.6	12.2	30.1	64.0	75.5
Others^g^	−2.0	−1.2	−2.9	−4.6	−5.5
**Net benefits**	**11.9**	**13.8**	**25.6**	**68.4**	**86.8**

^a^Results were calculated using mid-level economic parameters (detailed in Methods and Supplementary Data). Tables S6 and S7 provide the results obtained using low- and high-level economic parameters, respectively. ^b^Costs of large-scale crop farming mostly included those for land consolidation, along with the construction of ditches and field roads to meet the high cropland standards. ^c^Costs of large-scale livestock farming included construction expenses for building/renovating livestock farms and equipment investment. ^d^Benefits of manure management were primarily in the form of labor cost savings achieved by using an automatic manure scraper and sales of organic fertilizer. ^e^After implementing large-scale crop farming, cropland inputs were also reduced by efficiency gains, such as labor, machinery and services (except for the reduction in chemical fertilizer). ^f^After implementing large-scale livestock farming, the profit per unit livestock was greatly improved (e.g. from ¥163.93 to ¥413.69·head^−1^ for pigs). ^g^Negative values occurred because NH_3_ emission reductions can worsen acid rain.

Specifically, the costs of the three current traditional-farming-regime scenarios primarily originate from manure management and the use of enhanced-efficiency fertilizers. Furthermore, the main private benefits are derived from manure management—primarily selling organic fertilizer and the labor costs saved by using automatic manure scraper and manure application machines. After transformation to large-scale farming, the total costs of both the NMLF–PILOT and NMLF–ALL scenarios would increase by 2.5 and 3.9 times relative to those of NM, respectively. This increase is largely due to the huge investments necessary to implement cultivated land consolidation (US$11.3–19.4 billion) and construction/renovation of large-scale livestock farms (US$12.1–24.0 billion). However, these investments could yield substantial benefits to farmers and society over subsequent decades. The private benefits of NMLF–PILOT/ALL are 4.6–7.6 times greater than those of NM. This substantial increase is mainly attributed to reduced cropland area inputs due to efficiency gains, including labor, machinery and services, as well as profit expansion resulting from large-scale livestock farming. The societal benefits of NMLF–PILOT/ALL are also significantly higher than those of the three scenarios under the current traditional farming regime.

The most significant component of total benefits and societal benefits across all scenarios is the health benefit of reducing PM_2.5_. Health benefits can be valued using the statistical life value (US$250 000) and avoided premature deaths [31]. Under the current traditional farming regime, health benefits are only US$12.2–30.1 billion, but would increase to US$64.0–75.5 billion after large-scale farming implementation. Additionally, mitigation strategies would also reduce N_2_O and CH_4_ emissions, generating US$4.6–7.2 billion in GHG benefits under the large-scale farming regime.

## DISCUSSION

China's current annual PM_2.5_ level (30 μg·m^−3^ in 2023) greatly exceeds WHO guidelines. Controlling industrial emissions is increasingly difficult [32]; therefore, curbing agricultural NH_3_ has become critical for continued air quality mitigation. Previous studies likely overestimated NH_3_ mitigation potentials, as smallholder farming in China constrains the efficacy of the proposed strategies [17,33]. With large-scale farming gaining traction in China under supportive policies, we propose a feasible NH_3_ abatement strategy—integrating large-scale farming and N management—to achieve a 48%–58% reduction in NH_3_ emissions, avoiding 256–302 000 premature deaths attributed to PM_2.5_ exposure annually and yielding a net benefit of US$68.4–86.8 billion per year. Our findings could facilitate mitigation roadmaps not only for China but also other smallholder-dominated countries struggling to balance air quality and agricultural sustainability goals.

Under the current traditional farming regime, the maximum potential for NH_3_ reductions is only 30.9%, owing to the limited adoption of N management. Rapid urbanization has driven more young farmers to prioritize non-agricultural work and employ the simplest farming practices to maximize income; older farmers are less likely to embrace cutting-edge methods [17]. Substantial capital investments required for some NH_3_ mitigation technologies (e.g. ∼US$230 000 per anaerobic composting system) are also major obstacles for smallholder adoption of N management.

Although smallholder farming has been practiced in China for over 40 years, the challenges associated with smallholder farming, such as the abandonment of cultivated land and low mechanization levels, have become increasingly prominent [17,18]. The Chinese government has recognized the limitations of smallholder farming and has started to promote large-scale farming, investing substantial financial subsidies. Innovative institutional policies such as the *Administrative Measures for the Circulation of Rural Land Management Rights* [34], have enabled the separation of land ownership, contract rights and management rights, further greatly increasing the willingness of smallholders to transfer their farms to large-scale units. Since 2014, the area of cultivated land flowing into large-scale units has increased by 82.5%, reaching 16% of the total cultivated land area. Based on this development trend, we estimate that large-scale farming can be achieved in the pilot regions around 2035 and nationwide between 2050 and 2055. This time frame aligns with China's long-term agricultural development plans to essentially achieve agricultural modernization by 2035 and to fully achieve agricultural modernization and rural revitalization by the middle of this century [35].

Our findings show that NH_3_ mitigation potential under the current farming regime has limited effectiveness for improving air quality, resulting in only a 0.7–1.6 μg·m^−3^ national annual average PM_2.5_ decrease. China's most polluted areas often overlap with NH_3_ emission hotspots. In such NH_3_-rich regions, small reductions in NH_3_ emissions have little impact on PM_2.5_ levels [27,36]. The non-linear PM_2.5_–NH_3_ chemistry means that implementing substantial NH_3_ reductions is essential. Our proposed NH_3_ abatement strategy would achieve 9.4–14.0 μg·m^−3^ (up to 25.9 μg·m^−3^) PM_2.5_ decreases in heavily polluted regions. Concentration of NH_3_ emissions could lead to even greater PM_2.5_ benefit as more of that NH_3_ would be deposited locally before diluting to regional scales where PM_2.5_ formation is NH_3_ sensitive. It is worth noting that our model results show that further reductions in emissions of acidic gases, despite the associated challenges, can lead to additional improvements in air quality. For example, 20% reductions in emissions of NO_x_ and SO_2_ in addition to the NMLF–ALL scenario would result in additional 2.0% and 5.6% decreases in China's average PM_2.5_ concentrations in January and July, respectively (Table S8). However, substantial NH_3_ reduction plays a dominant role in lowering PM_2.5_ levels [11,36].

Our findings demonstrate that the proposed integrated strategy could realize triple benefits in increasing farmer incomes, ensuring food security and mitigating air pollution. Although promoting large-scale farming requires significant financial investment, with an average annual investment of US$19.4 billion for large-scale crop farming and US$24.0 billion for large-scale livestock farming, this policy can generate substantial returns. By improving agricultural production efficiency and the market competitiveness of crop and livestock products through superior management and mechanization, large-scale farming increases farmer revenues (e.g. 2.5-fold higher per pig profits) [37,38]. Our estimates show that in both the crop and livestock sectors, farmers’ incomes from large-scale farming exceed their costs (Table 2), and when combined with nitrogen management, even greater co-benefits can be obtained. Considering the practical difficulties and huge upfront investments required for implementing large-scale farming nationwide, our analysis suggests that the current pursuit of the NMLF–PILOT approach may be a more suitable option, as this scenario achieved maximum PM_2.5_ reduction efficiency and the highest benefit–cost ratio.

This study has some uncertainties. First, deposited NH_3_ may re-volatilize at high temperatures, which is not included in the GEOS-Chem model. Recent studies suggest that this process may influence the estimate of the impact of NH_3_ emissions on PM_2.5_ concentrations [39]. Second, the relatively coarse resolution of the GEOS-Chem model may result in a conservative estimate of PM_2.5_ reduction due to the heterogeneity of NH_3_ emissions under the large-scale farming scenario. Lastly, due to the high uncertainty associated with quantifying costs and private benefits across different food items, supply chain stages and regions [26,40,41], our analysis did not include these calculations for the RFLW scenario. All of these are the subjects of future studies. Despite these uncertainties, our proposed integrated strategies can help resolve China's dilemma of excessive agricultural NH_3_ emissions without effective control, substantially narrow the gap between current PM_2.5_ levels and WHO air quality guidelines, and promote agricultural sustainability.

## MATERIALS AND METHODS

### Large-scale farming prediction

Current farm size distribution in China was calculated using 30 × 30 m cropland data and field survey data [42] via the k-nearest neighbor machine-learning method [18]. Predicted farm size distribution following large-scale farming implementation was based on cropland spatial connectivity and terrain slopes (detailed in Text S2). N application rate reductions with farm size increase were evaluated according to statistical relationships derived from 863 000 field surveys across China [18,23,43]. Livestock production of large-scale farms under the current regime was allocated using real-world Amap POI data. With large-scale livestock farming, the free-range livestock was reallocated to large-scale farms within respective provinces. Additionally, the impact of selected thresholds used to define large-scale farming was also evaluated (see Text S2).

### NH_3_ emissions and mitigation scenarios

Gridded NH_3_ emissions were developed with 5’ × 5’ resolution [6]. Five NH_3_ emission reduction scenarios were developed: (i) using advanced technology solely (TECH, seven technologies selected, details in Text S3), (ii) reduced food loss and waste (RFLW), (iii) N management under current farming regime (NM, a combination of TECH and RFLW), (iv) N management throughout China following large-scale farming in pilot areas (NMLF–PILOT) and (v) N management after implementing large-scale farming throughout China (NMLF–ALL) (see Text S4 for details). The emissions reduction potential in each grid cell was calculated as:


(1) \begin{eqnarray*}
{{E}_r} &=& \left( {1 - \textit{adoption}} \right) \times E + \textit{adoption}\\
&& \times \, E \times \left( {1 - \eta } \right),
\end{eqnarray*}

where *E* and *E_r_* are the NH_3_ emissions pre- and post-N-management implementation, and *η* is the mitigation efficiency of specific advanced technologies. The combination of two or three mitigation technologies were calculated based on Zhang *et al.* [14]. *Adoption* is the adoption rates of advanced N management technologies for smallholder and large-scale farms, which were obtained from an extensive literature review of 18 967 farm surveys (see Text S5).

### Air quality simulations and PM_2.5_-attributable health burden

NH_3_, PM_2.5_ and associated species were simulated using the nested version of GEOS-Chem global CTM v.13.4.1 at 0.5° × 0.625° resolution (see Text S6). To assess the potential underestimation of PM_2.5_ reduction in the GEOS-Chem model due to the transition of livestock production to point sources under large-scale farming, we independently applied a box model for evaluation (see Text S6). Premature deaths from PM_2.5_ exposure were estimated using GEMM for chronic obstructive pulmonary disease, lung cancer, ischemic heart disease and stroke [44] (see Text S7).

### Cost–benefit analysis

We evaluated the abatement costs, private benefits and societal benefits of different NH_3_ mitigation strategies (see Text S8 for details). The net benefit was estimated as follows:


(2) \begin{eqnarray*}
Net &=& {{B}_{{societal}}} + {{B}_{{private}}}\\
&& -\, \left( {I*\frac{{{{{(1 + r)}}^{lt}}*r}}{{{{{(1 + r)}}^{lt}} - 1}} + FVO} \right),
\end{eqnarray*}

where *B_societal_* is the societal benefit, considered as the sum of benefits to human health, ecosystem health and GHG mitigation, minus the economic loss from reduced forestry and crop yields induced by NH_3_ reduction, and *B_private_* is the private benefits (e.g. labor-saving and increased profits). *I* is the total investment cost, *lt* is the abatement technique lifetime, *r* is the discount rate and *FVO* denotes annual fixed and variable operating costs.

## Supplementary Material

nwae324_Supplemental_File

## Data Availability

The ammonia emission inventories developed in this study are publicly available on Zenodo: https://doi.org/10.5281/zenodo.11218291.
