# Supplementary material for: Unlocking nitrogen management potential via large-scale farming for air quality and substantial co-benefits
Source: Natl Sci Rev. 2024 Sep 13;11(10):nwae324. doi: 10.1093/nsr/nwae324 (PMC11495489; doi:10.1093/nsr/nwae324)
Supplement: nwae324_Supplemental_File [file nwae324_supplemental_file.docx]

Supporting Information for

**Unlocking Nitrogen Management Potential via Large-scale Farming for Air Quality and Substantial Co-Benefits**

Baojie Li^1^, Hong Liao^1*^, Ke Li^1^, Ye Wang^1^, Lin Zhang^2^, Yixin Guo^3^, Lei Liu^4^, Jingyi Li^1^, Jianbing Jin^1^, Yang Yang^1^, Cheng Gong^5^, Teng Wang^6^, Weishou Shen^1^, Pinya Wang^1^, Ruijun Dang^7^, Kaihua Liao^8^, Qing Zhu^8^ and Daniel J. Jacob^7^

^1^ Collaborative Innovation Center of Atmospheric Environment and Equipment Technology, Jiangsu Key Laboratory of Atmospheric Environment Monitoring and Pollution Control, School of Environmental Science and Engineering, Nanjing University of Information Science & Technology, Nanjing 210044, China.

^2^ Laboratory for Climate and Ocean-Atmosphere Studies, Department of Atmospheric and Oceanic Sciences, School of Physics, Peking University, Beijing 100871, China.

^3^ Earth, Ocean and Atmospheric Sciences Thrust, Function Hub, Hong Kong University of Science & Technology (Guangzhou), Guangzhou 511442, China.

^4^ College of Earth and Environmental Sciences, Lanzhou University, Lanzhou 730000, China.

^5^ Department Biogeochemical Signals, Max Planck Institute for Biogeochemistry, Jena 07745, Germany.

^6^ College of Oceanography, Hohai University, Nanjing 210024, China.

^7^ John A. Paulson School of Engineering and Applied Sciences, Harvard University, Cambridge, MA 02138, USA.

^8^ Key Laboratory of Watershed Geographic Sciences, Nanjing Institute of Geography and Limnology, Chinese Academy of Sciences, Nanjing 210008, China.

*Corresponding Author. e-mail: hongliao@nuist.edu.cn

**This document includes:**

Supporting text

Figures S1 to S10

Tables S1 to S9

SI References

**Text S1. Estimation of NH_3_ emissions and comparisons with previous studies**

The gridded NH_3_ emissions in 2016 were calculated based on our previous study [1], and maintained a 5’ × 5’ resolution over mainland China. The relative stability of national NH_3_ emissions over the past two decades, with a coefficient of variation of merely 4% since 2000, supports the use of 2016 as a representative baseline year for current emission levels. A total of 50 sources were considered, including livestock waste, fertilizer application, synthetic ammonia, transportation, and biomass burning. The detailed emission sources are listed in the Table S3. The gridded NH_3_ emissions were calculated according as follows:

$E_{NH_{3}}=\sum_{i} \sum_{j} \sum_{k} A_{i,j,k}\times EF_{i,j,k}$ (S1)

where *i*, *j*, and *k* represent the specific NH_3_ emission sources, grid, and month, respectively; *A* refers to the activity level, such as the livestock excretion amount per unit, biomass burning amount, or motor vehicle mileage; and *EF* is the emission factor for each source.

The NH_3_ emissions inventory established here improved on previous EF estimates of livestock waste and fertilizer application by considering the effects of soil properties, fertilizer types, temperature, and precipitation [1,2]. The dates and times of fertilizer application were also comprehensively accounted for improving the corresponding estimation of monthly NH_3_ emission distributions. Additionally, the activity levels of fertilizer application were refined to county-level data (application amounts of each fertilizer and each crop in each county) from provincial-level data in order to more accurately derive NH_3_ emissions.

The uncertainty in NH_3_ emissions was calculated via the Monte Carlo method, which has been used extensively in various inventory studies [1,3,4]. Emission uncertainty can originate from the activity data and emission factors (EFs, including correction parameters). In terms of activity levels, nationally published statistics, including crop area, chemical industry production, and car ownership, among others, are typically accurate, and maintain small coefficients of variation (CVs; 5%–10%). For predicted parameters, such as rearing period, and fertilizer application rate, among others, larger CVs are produced (≥ 25%). Comparatively, the EFs for NH_3_ exhibited larger fluctuations (CVs ≥ 50%). Some local parameters used in the EF correction process (e.g., pH, cation exchange capacity) produced the largest CVs (~100%). It was assumed that all uncertainties in the activity levels and emission factors were normally distributed. The detailed parameters of the CVs for the two datasets were derived from Huang et al. [4] and Li et al. [1]. All NH_3_ emission calculations were replicated 10,000 times using a random selection of all inputs to estimate the 90% confidence intervals for the range of emissions.

Based on updated data, the calculated NH_3_ emissions in this study (mean, 12.84 Tg; 90% confidence interval range, 11.14–15.97 Tg) were slightly higher than those of our previous evaluation (12.11 Tg) [1]. Notably, the emissions found in the present study were similar to improved emission estimates based on AMoN-China NH_3_ observations and an ensemble Kalman filter (13.1 Tg) [5], and relatively close to bottom-up emissions calculated by Zhang et al. [2] (11.7 Tg), and Regional Emission inventory in Asia (REASv3) estimates (14.1 Tg) [6].

**Text S2. Large-scale farming prediction**

The current farm size distribution in China was calculated using cropland distribution data combined with the field survey data of Lesiv et al. [7]. High spatial resolution (30 × 30 m) cropland data (FROM-GLC2017) were obtained from http://data.ess.tsinghua.edu.cn. We converted the 30-m land use data into 1-km resolution point-grid data, and used the k-nearest neighbor machine learning method (k = 5) to predict farm sizes at unknown points [8], achieving a prediction accuracy of 79.2%. The distribution of predicted farm sizes following the implementation of large-scale farming was calculated according to the spatial connectivity and slope of the cultivated land. Specifically, we calculated terrain slopes of croplands using high-resolution DEM data [9]. Areas with slopes > 6° were considered unsuitable for large-scale farming. In the remaining cropland areas, we assumed that if multiple cropland cells were connected in space, the area they formed could create a single large-scale cropland plot [8]. Lastly, by splitting these plots using city administrative boundaries, the predicted farm size distribution following the implementation of large-scale farming was obtained.

Under the current smallholder crop farming regime, farmers tend to apply excessive chemical fertilizers to ensure yield; thus, farm size is a strong influential factor for fertilizer application rates, as confirmed by the statistical relationship with farm size obtained from 863,000 field surveys across China [10]. Accordingly, we evaluated reductions in N application rates with farm size increase as follows [8,11]:

$N_{LSF}=N_{Current}\cdot\exp\left[ \alpha\cdot\ln(\frac{FS_{Current}}{FS_{LSF}}) \right]$ (S2)

where *N_current_* and *N_LSF_* represent the N application rates of each crop type in each grid pre- and post-implementation of large-scale crop farming, respectively; *FS* represents farm size (ha); and *α* is the coefficient of large-scale farming, which was set to 0.264 based on Duan et al. [8]. When farm size becomes exceptionally large, the N application rates does not consistently decrease with the expansion of farm size. Therefore, based on a previous study, we assumed that the N application rate remains constant when the farm size exceeds 16 ha [8]. The spatial distribution of N application rates in 2016 was quantified based on county-level official N application rates data and high-resolution cropland distribution data.

Across the livestock sector, animals are raised under different systems, including free-range, large-scale, and grazing systems. We converted all livestock numbers to pig units, which allowed us to compare nitrogen (phosphorus) excretions from different types of livestock. One head of dairy cattle, beef cattle, sheep/goats, layer poultry, and broiler poultry are equivalent to 10, 5, 1/3, 1/15, and 1/60 pig units, respectively. The livestock production of large-scale farms under the current traditional regime was allocated based on their real-world locations obtained from Amap Map (a leading web mapping service in China, https://lbs.amap.com/api/) POI data. With large-scale livestock farming, the free-range livestock was reallocated to large-scale farms within respective provinces. The grazing livestock maintained this feeding strategy. Manure N emissions under the two regimes were calculated based on Huang et al. [4].

In addition, we evaluated the impact of selected thresholds used to define large-scale farming, and found it is relatively small. By varying the large-scale crop farming threshold between 1-3 ha, we found that it would only affect 6.1% of the total cultivated land area. Similarly, for large-scale livestock farming, we used cattle as an example. Varying the threshold between 100-500 heads would only influence 5.6% of the total cattle population.

**Text S3. Ammonia abatement technologies**

Although there are > 20 technical mitigation options for crop and livestock systems [12], the applicability of some technologies is limited by their efficacy or practical constraints. Seven technologies were selected based on their mitigation efficiency, practical limitations, and cost-effectiveness.

For crop systems, reducing N fertilizer overuse, using enhanced-efficiency fertilizers, and deep placement of N fertilizers were considered. The N application rate in China is nearly three times the global average [13]. This excessive N input leads to a decline in N use efficiency, and volatilization of NH_3_. Those with the highest NH_3_ emissions, namely maize, rice, wheat, and vegetables, were examined. The reduction ratios of maize, rice, and wheat were determined using the methods of Guo et al. [14]. China was divided into 7–13 regions for maize, rice, and wheat, for which each region was characterized by a different proportion of N fertilizer reduction. For vegetables, we used the recommended N application rates (195–345 kg N·ha^-1^) at the provincial-scale (Table S9).

Enhanced-efficiency fertilizers included nitrification inhibitors, urease inhibitors, and controlled-release fertilizers. Nitrification inhibitors can decrease N_2_O emissions at the expense of increased NH_3_ emissions; therefore, only the two other types of enhanced-efficiency fertilizers were considered here. We calculated the average emission reduction efficiency of the two fertilizers for maize, rice, and wheat using corresponding emission reduction data from the meta-analysis of Ti et al. [15]. Control-release fertilizers have a greater ammonia reduction effect on maize and rice; whereas urease inhibitors are correlated with a greater ammonia reduction effect on wheat. The resulting emission reduction effects using enhanced-efficiency fertilizer for maize, rice, and wheat were 41.2% ± 14.0%, 52.0% ± 33.7%, and 58.7% ± 32.9%, respectively [15].

The deep placement of N fertilizer can adsorb additional NH_4_^+^ via soil colloids and inhibit NH_3_ volatilization [15]; thus, it has proven to be a simple and effective emission-reduction technology. The adoption of deep fertilizer placement in China was 23.3% in 2016, notably similar to the adoption rate of advanced N management technologies by smallholder farms. Owing to the difficulty of deep N placement by machines over certain terrains (e.g., mountains), cultivated land with slopes > 6° were deemed unsuitable for deep N placement here.

For livestock systems, low crude protein feeding was considered because it can reduce protein intake and manure N excretion, while maintaining normal production levels. Overall, this method can reduce NH_3_ emissions by 10%–20% for pigs, poultry, and cattle [12]. Based on the actual conditions of farms and rearing livestock housing conditions, the use of bedding materials or frequent manure removal techniques can be adopted to reduce NH_3_ emissions by 20%–50% [12,16]. During manure storage, adding acidic additives, along with the use of anaerobic composting technology can reduce corresponding NH_3_ emissions by 60% [14,17]. Comparatively, different emission reduction strategies can be used for solid and liquid manure application, in which the incorporation of solid manure can reduce NH_3_ emissions by 45%–65%, and the injection of liquid manure can reduce emissions by 80%–90% across all livestock types [12].

**Text S4. Mitigation scenarios**

**Technology advanced (TECH) scenario.** For TECH, we considered only the adoption of advanced technologies under the current traditional regime, where smallholder crop and livestock farms are dominant. Although there are > 20 technical mitigation options for crop and livestock systems [12], the applicability of some technologies is limited by their efficacy or practical constraints. For example, applying soil amendments such as biochar and organic acids to improve soil fertility and reduce NH_3_ emissions is difficult owing to their high costs and wide variation of soil properties across China [12]. Ultimately, seven technologies were selected based on their mitigation efficiencies, practicality, and cost-effectiveness (details in Text S3).

**Reducing food loss and waste (RFLW) scenario***.* Each year, 27% of food produced in China for human consumption is lost or wasted post-harvest during storage, processing, and distribution [18]. Accordingly, RFLW is an effective consumption-side strategy to decrease NH_3_ emissions. The loss fractions of staple foods (including maize, rice, and wheat) and fruits/vegetables are 5.5% and 28%, respectively [18]. 50% of meat (pigs, sheep, poultry, cattle) is lost or wasted across the supply chain. Such losses cannot be entirely avoided. For example, fruits and vegetables are easily damaged in supermarkets by consumer activity. Even then, ~40%–60% of food losses can still be further mitigated [19]. Accordingly, we set the food loss reduction potential to 50% in the present study.

**Implementing N management under current traditional farming (NM)***.* The NM scenario was characterized by a combination of TECH and RFLW. NH_3_ mitigation via NM represents the maximum potential under the current smallholder-dominated farming regime.

**Implementing N management following large-scale farming in pilot areas (NMLF–PILOT) and throughout China (NMLF–ALL)*.*** Large-scale crop and livestock farming can lead to a sharp decline in NH_3_ emissions owing to high mechanization and advanced technology adoption levels. Considering the difficulties related to implementing large-scale crop and livestock farming (e.g., enormous upfront investments), two scenarios were developed in this study. (1) NMLF–PILOT scenario: in the pilot area, both large-scale farming and N management (a combination of TECH and RFLW) are implemented, whereas only N management is implemented in other areas. Based on NH_3_ emission and PM_2.5_ pollution hotspots, 10 provinces were selected as pilot areas for large-scale farming (Fig. 2a), accounting for 20.8% of the area in mainland China and 55% of the total NH_3_ emissions. (2) NMLF–ALL scenario: large-scale farming and N management are fully implemented throughout China. The advanced N management technologies adopted in these two scenarios are consistent with those in the TECH scenario. The specific calculation processes used for the large-scale crop and livestock farming scenarios are described in the “Large-scale farming prediction” section.

**Text S5.** **Advanced N management technology adoption under traditional and large-scale farming regimes**

The adoption rates for advanced N management technologies vary considerably for smallholder farming and large-scale farming regimes. Data on advanced technology adoption were obtained from an exhaustive literature survey of peer-reviewed publications using the ISI Web of Science and China Knowledge Resource Integrated databases. Survey data from four articles were collected in 2013–2015; whereas all remaining data from 28 articles were obtained from surveys conducted in or after 2016, supporting the applicability of the data to the present study [20–51]. Data on the adoption rates of advanced technologies were obtained for 6,546 smallholder crop farms (farm size ≤ 2 ha), 7,549 large-scale crop farms (farm size > 2 ha), and 4,872 large-scale livestock farms.

The adoption rates of advanced N management technologies for large-scale crop farms, smallholder crop farms, and large-scale livestock farms were 43.8%, 22.2%, and 39.1%, respectively (Fig. S2). Notably, the adoption rate of large-scale crop farms was nearly twice that of smallholder farms. Owing to limited survey results related to advanced technology adoption on smallholder livestock farms, the adoption of smallholder livestock farms was assumed as 19.6% based on the ratio of technology adoption rates between large-scale and smallholder crop farms. Furthermore, policy instruments, such as financial incentives, can facilitate the more rapid adoption of advanced technologies [52]. We assumed here that pursuing a more positive policy would double the adoption rate of advanced technologies based on the change in the installation rate of end-of-pipe equipment following the implementation of *Air Pollution Prevention and Control Action Plan* during 2013–2017 [53]; thus, the adoption values (*adoption* in equation (1) of the main text) of large-scale crop farms, smallholder crop farms, large-scale livestock farms, and smallholder livestock farms used set to 87.6%, 44.4%, 78.2%, and 39.1% in China, respectively.

**Text S6. Air quality simulations**

**GEOS-Chem simulations**. Simulations of NH_3_, PM_2.5_, and their associated species were performed using the nested version of GEOS-Chem global CTM *v.*13.4.1 (http://wiki.seas.harvard.edu/geos-chem/index.php/GEOS-Chem_versions#13.4). GEOS-Chem includes fully coupled ozone–NOx–VOC–aerosol chemistry [54,55]. The nested domain (68–140° E, 10–55° N) had a horizontal resolution of 0.5$^{\circ}$ latitude × 0.625$^{\circ}$ longitude, and contained 47 vertical layers. Concentrations at the lateral boundaries were provided by a global GEOS-Chem simulation at a horizontal resolution of 2$^{\circ} \times$ 2.5$^{\circ}$, and were updated every 3 h. Assimilated meteorological data from the 2016 NASA Modern-Era Retrospective Analysis for Research and Applications *v*.2 (MERRA-2) were used to drive the model, and each simulation had a 6-month spin-up. While NH_3_ emissions estimates were obtained directly from this study, other anthropogenic emissions of air pollutants (including SO_2_, NO_x_, CO, CO_2_, NMVOC, BC, OC, PM_2.5_, and PM_10_) in China were obtained from the 2016 Multi-resolution Emission Inventory for China (MEIC).

Aerosol simulations using GEOS-Chem have been extensively evaluated across China [56,57] and the world [54,55], showing strong consistency between observations and modeled results. We simulated the air quality under the five scenarios (TECH, RFLW, NM, NMLF–PILOT, and NMLF–ALL) to assess the impacts of different NH_3_ abatement scenarios on air quality. Furthermore, considering the challenges in further reduction of emissions of acidic gases in China, we designed an additional experiment in which NO_x_ and SO_2_ emissions were reduced by 20% in addition to the fully implemented NMLF–ALL strategy, to investigate the effect of coordinated reductions of NH_3_ and acidic gases on PM_2.5_.

A baseline simulation was conducted to evaluate model performance, and confirmed that the simulated PM_2.5_ values fit the measurement data well. We validated the GEOS-Chem simulation using the China Ministry of Ecology and Environment (MEE) surface measurement network. The daily observed and simulated PM_2.5_ concentrations were compared for the three main megacity clusters—BTH, SCB, and YRD—revealing correlation coefficients of 0.81, 0.68, and 0.83, respectively (Fig. S9). Furthermore, we compared NH_3_ vertical column densities (VCDs) derived from the infrared atmospheric sounding interferometer (IASI) satellite observations with those from GEOS-Chem simulations. Daily IASI NH_3_ VCDs were obtained from the ESPRI Data Center (https://cds-espri.ipsl.upmc.fr). High correlations between the simulated NH_3_ VCDs and IASI observations were found in the density scatterplot, with R^2^ values of 0.65, 0.84, 0.67, and 0.45 for spring, summer, autumn, and winter, respectively (Fig. S10).

**Box model simulations**. To assess potential underestimation of PM_2.5_ reduction in the GEOS-Chem model from transitioning livestock production to point sources after large-scale farming, we applied a box model version of the Community Multiscale Air Quality Modeling System (CMAQ). The box model employed in this study does not consider the bi-directional feedback between NH_3_ emission and NH_3_ deposition, which is consistent with the settings in GEOS-Chem. The model was run using meteorology and emissions representing average January 2016 conditions in the BTH region. The meteorological field was prepared based on eastern China predictions from the Weather Research and Forecasting model version 4.0 (WRF v4.0) constrained by European Centre for Medium-Range Weather Forecasts Reanalysis v5.0 (ERA5). Data on NH_3_ emissions were obtained from this study, while those for other pollutant species followed MEIC. Since livestock facilities concentrate NH_3_ dry deposition within 5 km, the box model resolution was set to 5km × 5km. A GEOS-Chem grid cell (~3,000 km^2^) was divided into 120 boxes of 5 × 5 km (Fig. S7). Without considering in-grid emission heterogeneity, the box model simulated a 14.8% decline in PM_2.5_ after implementing N management following large-scale farming, consistent with the 13.8% PM_2.5_ reduction simulated by GEOS-Chem across the BTH region in winter, demonstrating the reliability of the box model.

The box model was then applied to simulate three cases: livestock emissions within a 3000 km^2^ GEOS-Chem grid cell (1) uniformly distributed, (2) concentrated in 60 boxes (1500 km^2^), and (3) entirely concentrated into 1 box (25 km^2^), while keeping the NH_3_ emission densities from N fertilizer application and other sources unchanged (Fig. S7). We found that concentrating all livestock NH_3_ emissions into one box (Case 3) increased NH_3_ concentrations and dry deposition in that box by 178-fold and 93-fold, respectively, but only raised PM_2.5_ concentrations by 19.1% because of the NH_3_ excess, compared to the corresponding box in the uniform distribution case (Case 1). Consequently, despite consistent total livestock NH_3_ emissions on the scale of the GEOS-Chem grid cell, concentrating emissions increased average NH_3_ concentration and total NH_3_ dry deposition by 95.2% and 34.3%, respectively, while decreasing average PM_2.5_ concentration by 11.3%, implying that excessive localized NH_3_ makes PM_2.5_ formation less effective. With livestock emissions were allocated into 60 boxes instead (Case 2), the effect of the localized NH_3_-rich environment was significantly reduced, with grid cell average PM_2.5_ concentration changing by only −1.2% compared to the uniform distribution case (Case 1) (Fig. S7).

**Text S7. Public health impact of PM_2.5_**

Premature deaths from exposure to ambient PM_2.5_ exposure were estimated based on the methods of the Global Burden of Disease (GBD) [58]. The four diseases considered in this study were chronic obstructive pulmonary disease, lung cancer, ischemic heart disease, and stroke. The attributable premature mortality rates from PM_2.5_ exposure were then quantified using the newly developed Global Exposure Mortality Model (GEMM) [14,59,60]. Premature death (*M*) due to each disease (*i*) was calculated as:

$M_{i,g}=Pop_{g}\times Rate_{a}\times Mortbase_{a,i}\times(1-\frac{1}{RR_{a,i,g}})$ (S3)

where *Pop* is the total population in each 0.625º× 0.5º grid (*g*), as was obtained through the calibration of Landscan data (https://landscan.ornl.gov/) and provincial official statistics; *Rate_a_* represents the proportion of population in each age group (*a*); *Mortbase_a,i_* is the baseline mortality of each disease in each age group (*a*) (https://vizhub.healthdata.org/gbd-results/); and *RR* is the relative risk of noncommunicable diseases and lower respiratory infections evaluated in the GEMM, values which are specific to each age group for IHD and stroke. A total of 12 age groups were considered: 25–29, 30–34, 35–39, 40–44, 45–49, 50–54, 55–59, 60–64, 65–69, 70–74, 75–79, and > 80 years.

**Text S8. Estimating cost–benefits of different NH_3_ mitigation scenarios**

We evaluated the abatement costs, private benefits, and societal benefits of different NH_3_ mitigation strategies. The net benefit was estimated as follows:

$Net=(B_{HH}+B_{EH}+B_{GHG}-AR_{damage}+B_{private})-(I*\frac{(1+r)^{lt}*r}{(1+r)^{lt}-1}+FVO)$ (S4)

where *B_HH_*, *B_EH_*, and *B_GHG_* are the benefits to human health, ecosystem health, and GHG mitigation, respectively; *AR_damage_* induced by NH_3_ reduction refers to the economic loss from reduced forestry and crop yields, as evaluated by Liu et al. [61]; and *B_private_* primarily includes savings from the reduced use of N fertilizer, labor, additional income from organic fertilizer sales [14], improved profits after changing farming methods from free-range to large-scale farming [62], and savings by efficiency gains with the farm size increase [8]. The annual implementation cost was calculated as the sum of annual investments, fixed operating costs, and variable operating costs [16], where *I* in eq. (S4) is to the total investment cost, *lt* is the lifetime of the abatement technique, *r* is the discount rate, and FVO is the annual fixed and variable operating costs (e.g., labor, electricity). The investment costs included both those for purchasing equipment (e.g., automatic manure scraper), cultivated land consolidation, and construction of building/renovating large-scale livestock farms in the NMLF–PILOT and NMLF–ALL scenarios.

The societal benefit was considered as the sum of the *B_HH_*, *B_EH_*, and *B_GHG_*, minus *AR_damage_*. The health benefits to human health (*B_HH_* in eq. (S4)) were evaluated as follows:

$B_{HH}=M\times VSL$ (S5)

where *M* is premature death, and *VSL* is the value of statistical life. We adopted the latest statistical life value of ~US$ 250,000 using the willingness-to-pay method in China [63]. Ecosystem beneﬁts (B_EH_) were regarded as the avoided damage costs of ecosystem eutrophication due to NH_3_ mitigation and were obtained from the European Nitrogen Assessment [12,61,64]. The GHG benefit was considered as the avoided abatement costs of GHG emissions, and was obtained as follows:

$B_{GHG}=MAC_{GHG}\times\Delta E_{GHG}$ (S6)

where *ΔE_GHG_* is the corresponding reduction in GHG emissions, which used 34 kg CO_2_-eq for CH_4_ emissions and 298 kg CO_2_-eq for N_2_O emissions across a 100-year time scale [12,65]; and *MAC_GHG_* represents the marginal abatement costs (carbon price) to reduce 1 t of GHG emissions in US$ per ton CO_2_-eq (10–50 US$) [14,66]. The effects of NH_3_ control technology on GHG emissions were obtained from Zhang et al. [12]. We calculated the costs and benefits under low-, medium-, and high-level economic parameters.

**TECH scenario.** For croplands, the cost of reducing the overuse of N fertilizer is nil, which also leads to reduced fertilizer purchase expenses. Here, we estimated the costs of current N use by assuming an average N price for farmers’ current N use mix (urea and compound fertilizer) equal to the price of urea N (¥3·kg^-1^ N). Notably, the use of enhanced-efficiency fertilizers would lead to increased costs. According to a brief survey of China’s largest shopping websites (https://www.taobao.com/ and https://www.1688.com/), the prices of slow-release fertilizer and N-(n-butyl) thiophosphoric triamide (NBPT) urease inhibitor are ¥8·kg^-1^ N (¥5.6–10.5·kg^-1^ N) and ¥120·kg^-1^ (¥100–140·kg^-1^), respectively. NBPT urease inhibitor is applied 1,200 mg·kg^-1^ urea. The costs of renting machines for deep application of chemical fertilizer were estimated as ¥375·ha^-1^ (¥225–525·ha^-1^). We then calculated the crop area of deep N fertilizer placement for different regions to obtain the total costs of machine rental in China.

In the livestock sector, low crude protein feeding can be applied to pigs, cattle, and poultry to save a feed cost of ¥9–106·t^-1^ [67]. Current feed costs of different livestock types for free-range and large-scale farming were obtained from the *China Agricultural Products Cost-Beneﬁt Yearbook* [62]. After considering the differences in advanced technology adoption between traditional and large-scale farming regimes, the cost saved by low-crude protein feeding was calculated.

Based on the actual conditions of farms and rearing livestock housing, bedding materials or frequent manure removal techniques can be adopted to reduce NH_3_ emissions by 20%–50%. In this study, the costs of frequent manure removal techniques were estimated. Although the implementation of this technique incurs investment costs, it is generally cost-effective, as frequent manure removal can help farmers efficiently complete manure-cleaning, thereby reducing labor costs. We investigated the cost of automatic manure scrapers in cattle, pig, and poultry farms through the largest online shopping website in China (https://www.taobao.com/), revealing prices of ¥8000–16000·set^-1^, ¥6200–8800·set^-1^, and ¥4120–8000·set^-1^, respectively. The lifetime of each set is approximately 8 years, while each manure scraper must be maintained every 3 years, at a cost of ¥3000·set^-1^. Lastly, the total annual costs of frequent manure removal technique use for cattle, pig, and poultry farms were ¥32.7–56.4·head^-1^, ¥4.1–5.3·head^-1^, and ¥0.10–0.17·head^-1^, respectively. As it takes an average worker 0.9–1.6 h to clean 1 t of feces, the labor costs saved per the amount of manure emissions and price of workers were calculated based on an average worker’s salary of ¥10·h^-1^ [62].

Furthermore, the use of acidic additives and anaerobic composting technology can significantly reduce NH_3_ emissions during manure storage. For large-scale farming, acidification of manure and anaerobic composting reactors are used for all indoor animals. Here, the calculation method was obtained from Guo et al. [14], For free-range farming, farmers can reduce NH_3_ emissions through the addition of acidic additives or cationic adsorbents. Zeolite (¥500–900·t^-1^, www.taobao.com) can be used to lower manure pH at application rates of 100 g·kg^-1^ manure [68]. For manure application estimation, we adopted a calculation method similar to that for the deep application of chemical fertilizers, basing costs on machine rental for injection and incorporation.

**RFLW and NM scenarios.** Food loss and waste occur at various stages of the food supply chain, including production, postharvest handling and storage, processing, distribution, retailing, and consumption [18], and involve a wide range of stakeholders and multiple dimensions, including quantity, environment, nutrition, and economics. The costs and benefits at each stage vary significantly for different food items, and data availability remains a major challenge [69,70]. Considering the substantial uncertainties in cost-benefit analyses, we did not calculate the costs and private benefits of RFLW. The societal benefits of RFLW were evaluated using eq. (S4).

The implementation of agricultural nitrogen management under the traditional farming regime (NM) was derived via the combination of TECH and RFLW. The calculation methods of cost–benefits were identical to those of TECH; however, the cost–benefit values were distinct from TECH, as the reduction in food waste and loss decreases the planting area of main crops (maize, wheat, rice, and vegetables) and manure amounts in the livestock sector.

**NMLF–PILOT and NMLF–ALL scenarios.** Cost analyses for implementing N management across China after transitioning to large-scale farming in the pilot areas (NMLF–PILOT) and throughout China (NMLF–ALL) included the enormous investments of transforming smallholdings into large-scale farms. For crop farming, land consolidation is essential to achieve large-scale farming, and its regional costs vary owing to different altitude and socioeconomic conditions. Here, we calculated the total land consolidation costs based on the unit cost ($2,634–3,787 USD·ha^-1^) of 201 land consolidation projects according to Duan et al. [8]. It was assumed that large-scale farming could be sustained for 50 years following consolidation; therefore, the lifetime of the abatement technique (*lt*) in eq. (S4) was equal to 50. Although the upfront, one-time financial investment is substantial, it can greatly reduce the use and corresponding costs of N fertilizer. Other inputs to cropland areas, including labor, machinery, and services, could also be reduced by efficiency gains from increased farm size. The additional private benefits of large-scale farming were obtained from Duan et al. [8].

In the livestock sector, building/renovating a considerable number of large-scale farms for the conversion of various free-range breeding types is necessary. Owing to the difficulty in estimating the construction costs of renovating an existing livestock farm, which includes upgrading equipment, demolishing houses, etc., we assume that the costs of renovating an existing large-scale livestock farm is equal to the costs of constructing a new large-scale livestock farm. Here, we obtained the total construction costs of pig, cattle, and chicken farms from our survey. Construction costs primarily included land leases, civil construction, equipment (e.g., water and power supply, sewage treatment), and transportation facilities. A civil construction service life of 30 years was assumed; whereas the lifetime of all other equipment was considered to be 10 years. The evaluated annual investment costs of cow, beef cattle, pig, and laying hen farms were ¥1000–2500, ¥400–900, ¥60–200, and ¥4.8–17.5·year-end head^-1^, while that of broiler farms was ¥0.2–0.6·slaughtered head^-1^. The profits from large-scale and free-range farming were obtained from the *China Agricultural Products Cost-Beneﬁt Yearbook* [62]*.* Following transformation to large-scale farming, farmers’ profits can be substantially improved. For example, the profits of pig farms through free-range and large-scale farming are ¥163.93 and 413.69·head^-1^, respectively [62], where improved profits are primarily derived from a sharp reduction in labor costs.

Except for the deep application of N fertilizer and manure, the cost–benefit calculations of all other technologies under NMLF–PILOT and NMLF–ALL were consistent with those of TECH. While rental machines were used in the TECH scenario for the deep application of N fertilizer and manure, farmers engaged in large-scale farming are more inclined to reduce NH_3_ emissions by purchasing machines. The machine price for deep fertilizer application is ¥20,000–35,000·set^-1^ (https://b2b.baidu.com/), and was estimated to have a service life of 10 years. The annual fixed and variable operating costs (FVO) per machine were derived from official statistics [62]. We then calculated the annual investment cost of deep application as ¥23.4 (¥21.0–24.7) ·ha^-1^. Additionally, the price of machines for manure application was estimated as ¥200,000–300,000·set^-1^ (https://b2b.baidu.com/), with a service life of 10 years. Each machine can apply 15,000 t manure per year. The FVO was consistent with deep fertilizer application.


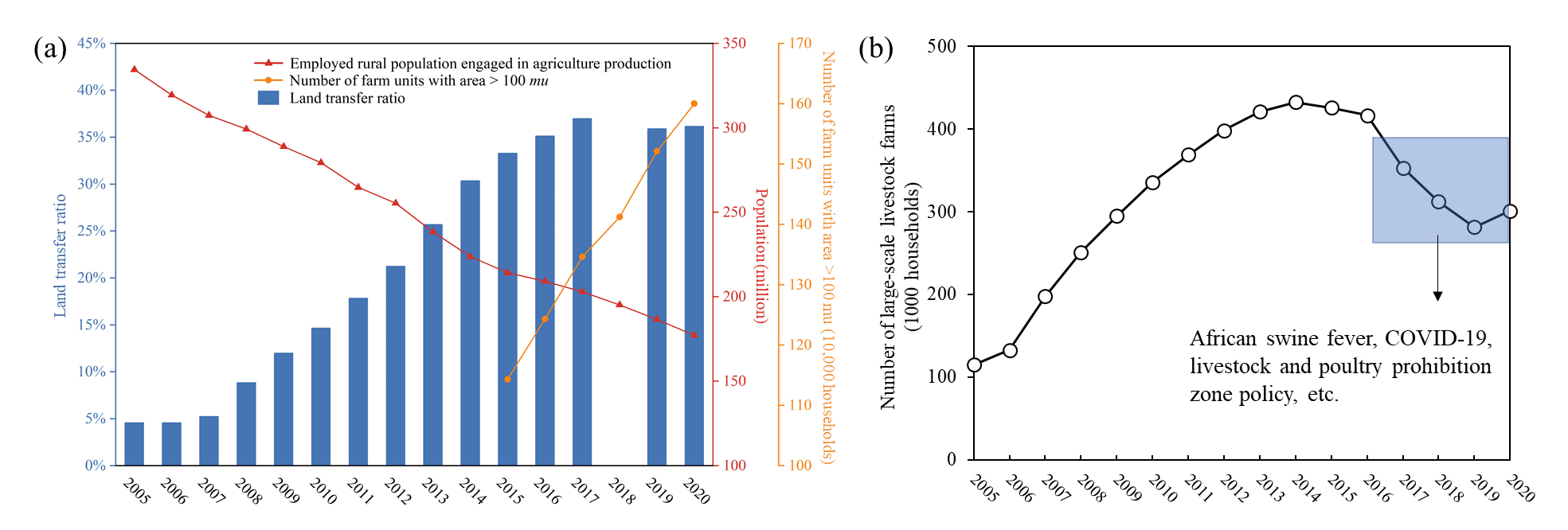


Fig. S1. Ongoing trend of large-scale farming. (a) Changes in land transfer ratio, employed rural population engaged in agriculture production, and number of farms with an area > 100 mu (6.7 ha) from 2005–2020. (b) Change in number of large-scale livestock farms in China. Here, 1 ha is equal to 15 *mu*.


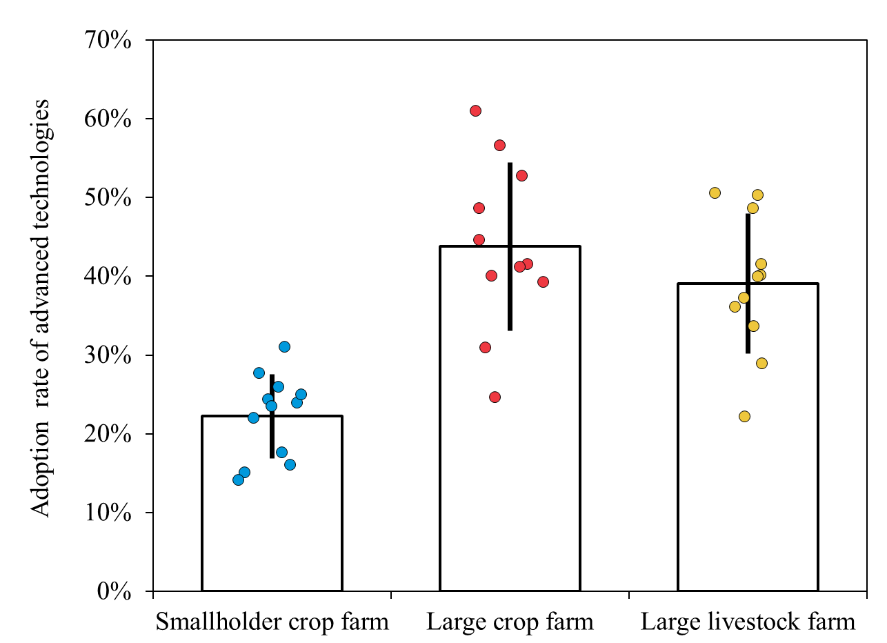


Fig. S2. Advanced N management technology adoption rates of smallholder crop farms, large-scale crop farms, and large-scale livestock farms across China.

**
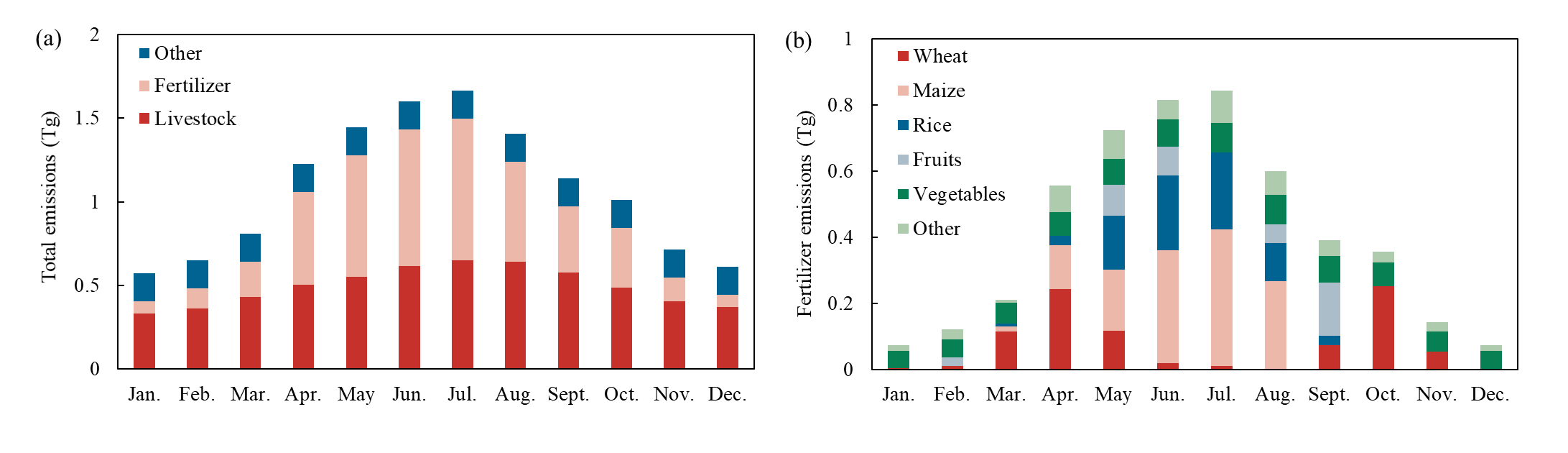
**

**Fig. S3. Monthly NH_3_ emissions**. (a) Fertilizer application, livestock waste, and other; (b) fertilizer application from different crops.


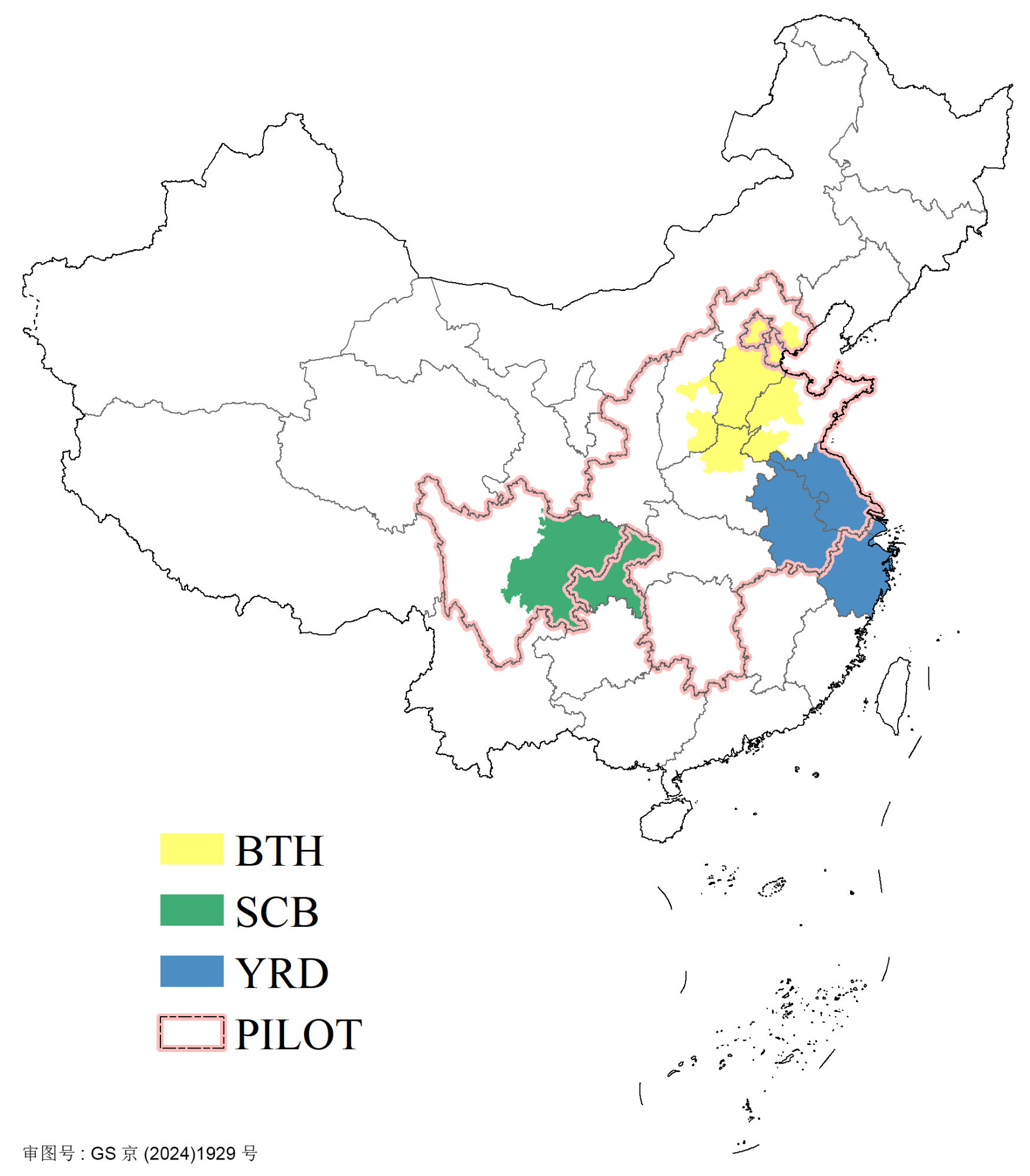


**Fig. S4. Location of PILOT, BTH, SCB, and YRD in China.** BTH referred to the “2+26” (Beijing, Tianjin, and 26 other municipalities in the surrounding area) region. YRD and SCB are the Yangtze River Delta and Sichuan Basin, respectively. PILOT represents the 10 provinces with the highest concentrations of PM_2.5_ and NH_3_ emissions, which account for 20.8% of China’s total land area. Data from the Hong Kong, Macao, and Taiwan are not available in this study.

**
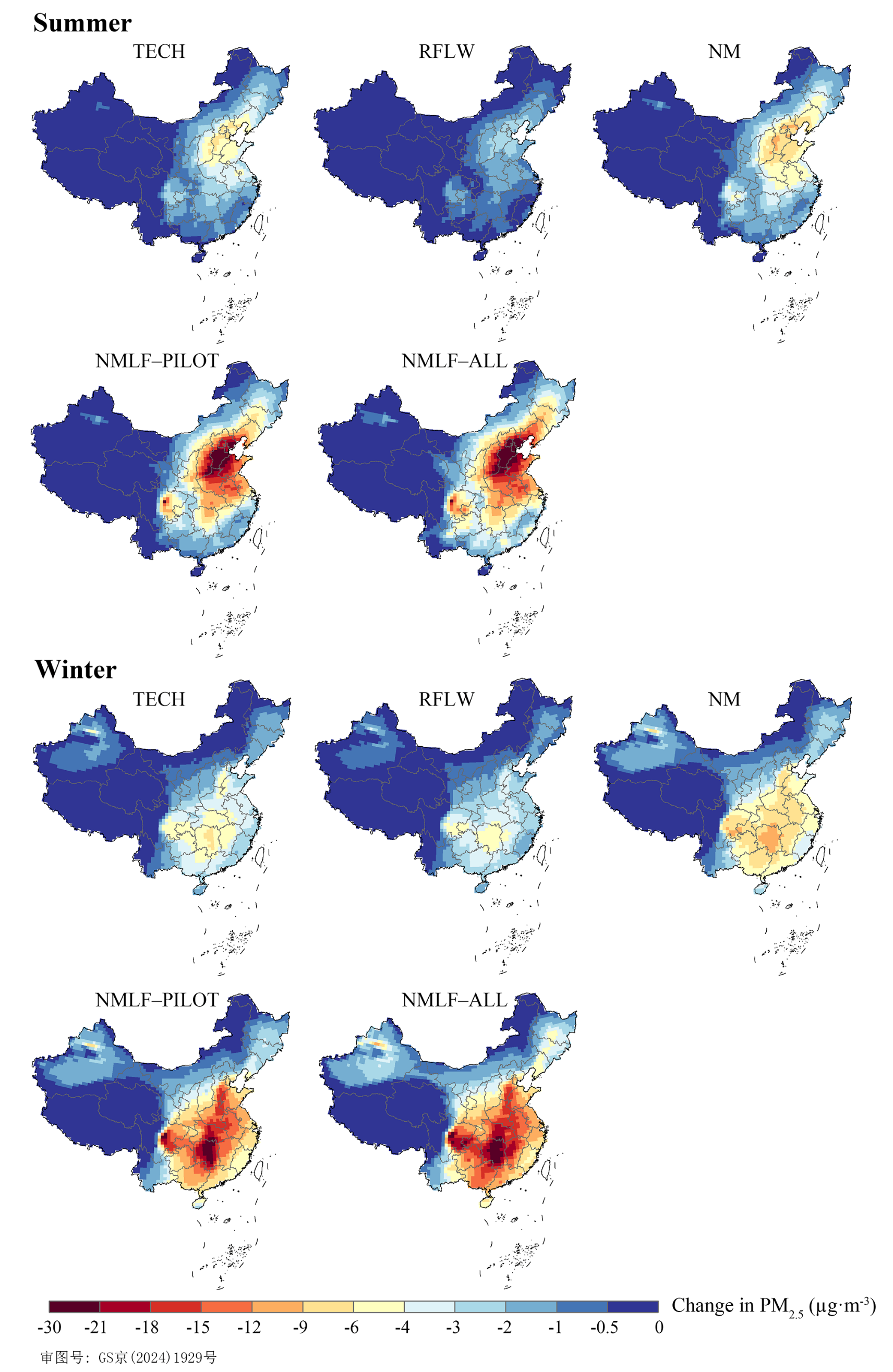
**

**Fig. S5. Changes in ground-level PM_2.5_ concentration under the five scenarios compared with the baseline simulation.** Values were calculated using the GEOS-Chem model with a 0.625º × 0.5º grid for the summer and winter of 2016. Data from the Hong Kong, Macao, and Taiwan are not available in this study.


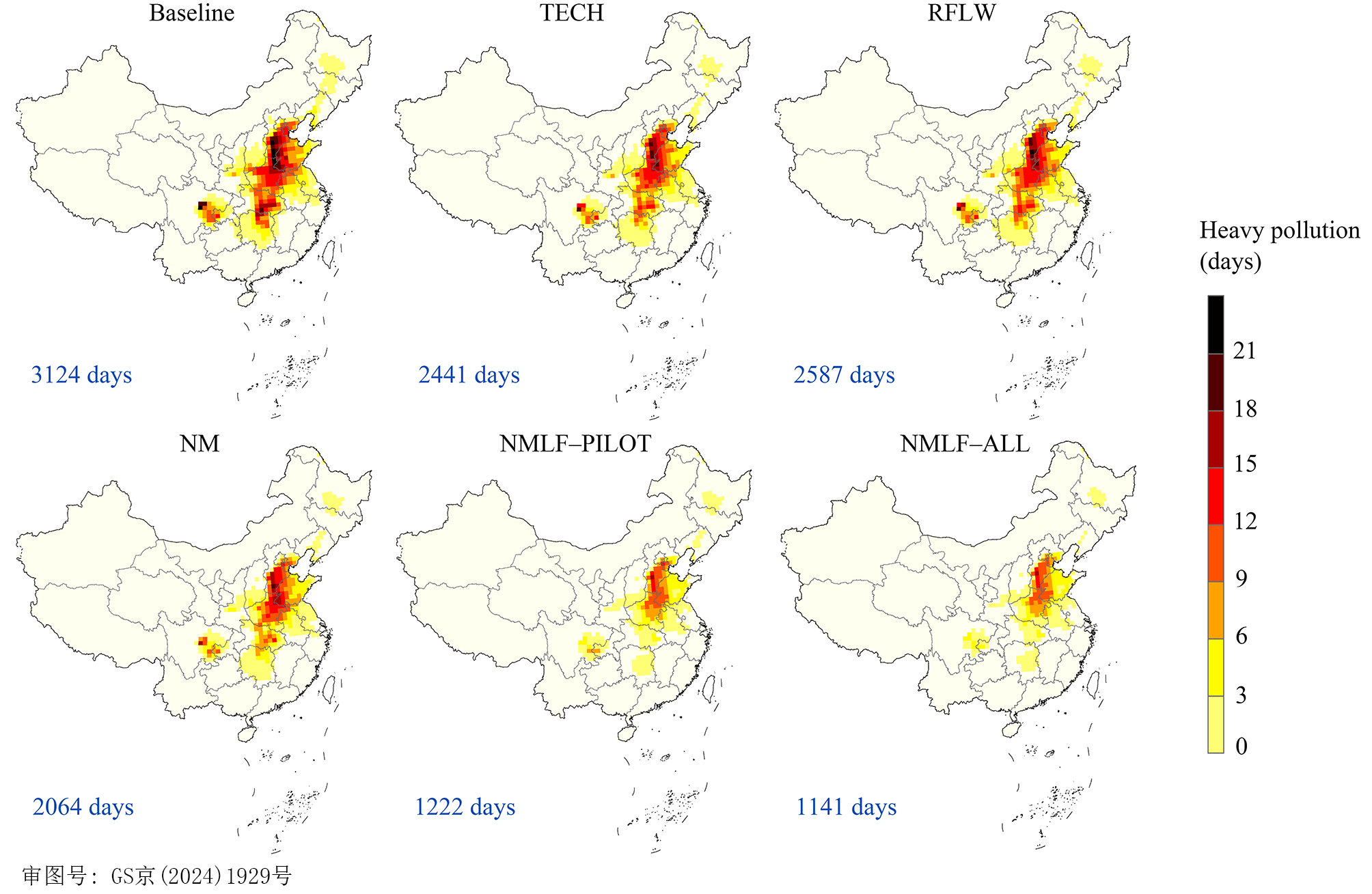


**Fig. S6. Spatial distribution of heavy pollution days under the baseline scenario and five emission reduction scenarios.** Data from the Hong Kong, Macao, and Taiwan are not available in this study.


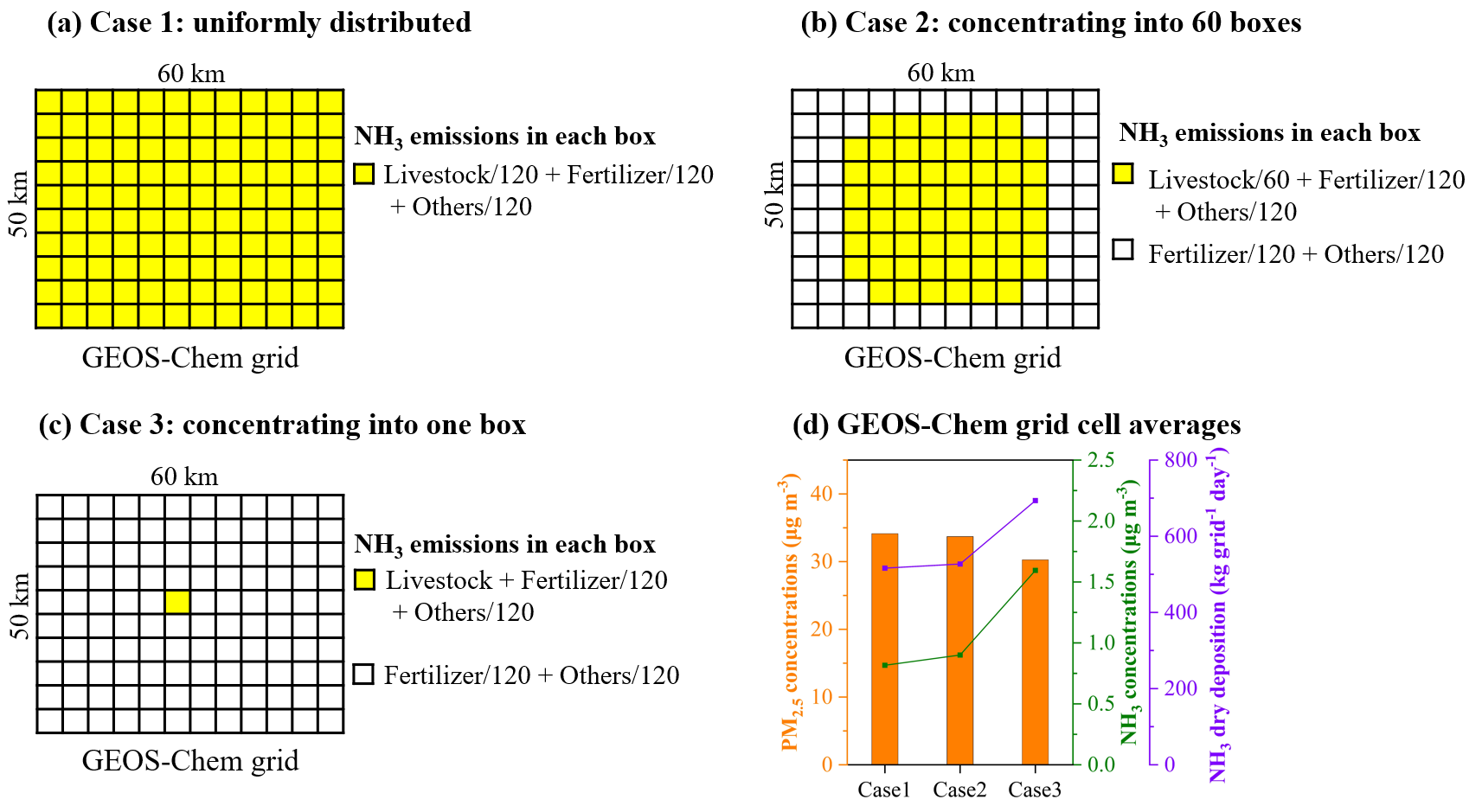


**Fig. S7. Three cases applied in the box model and their related results.** Three cases: livestock emissions within the 3,000 km^2^ grid (a) distributed uniformly into each box, (b) concentrated into 60 boxes, (c) entirely concentrated into 1 box, and (d) the average PM_2.5_ and NH_3_ concentrations, and the total NH_3_ dry deposition fluxes on the scale of the GEOS-Chem grid cell simulated by the box model.


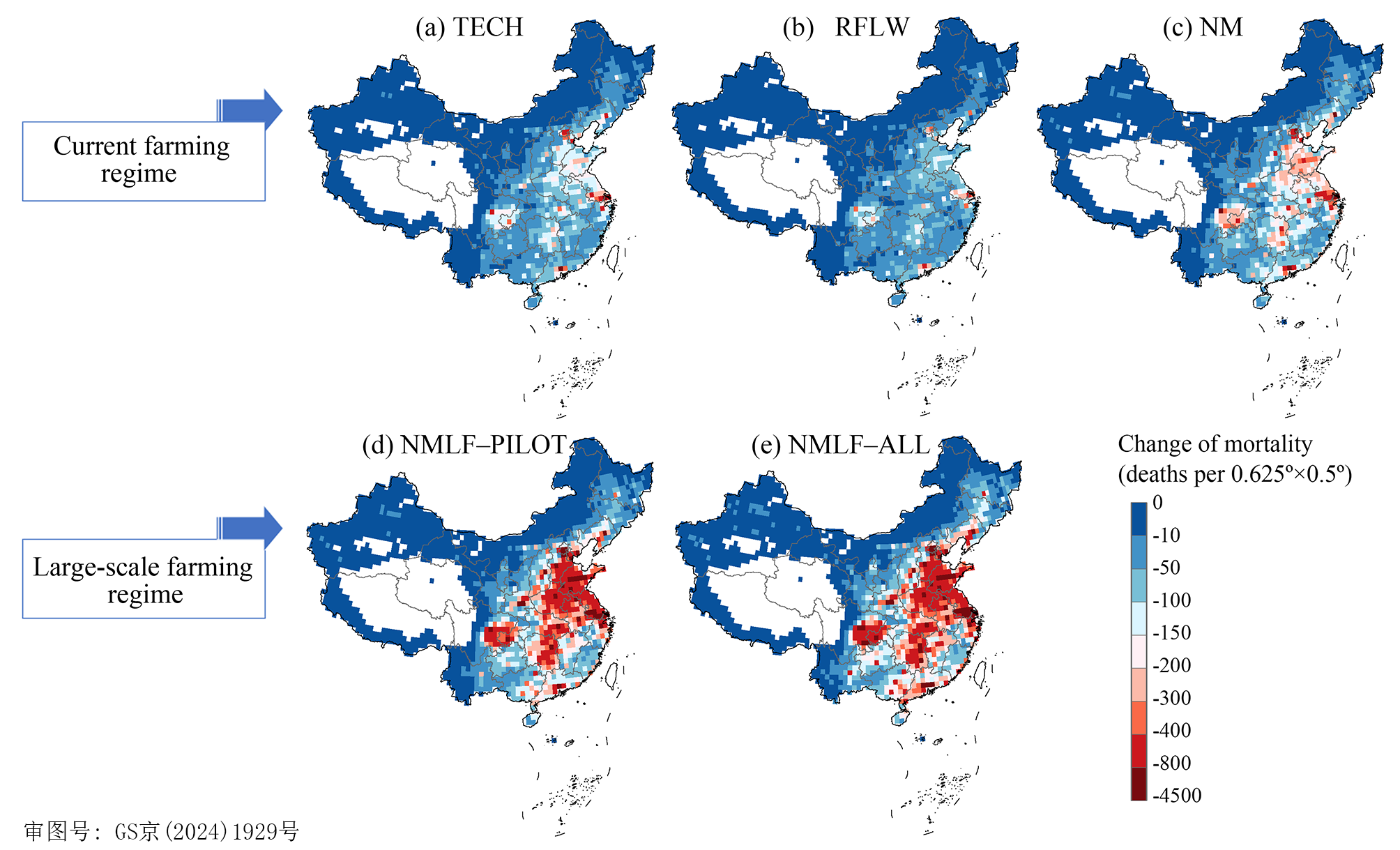


**Fig. S8. Health burden impacts based on the implementation of five different scenarios.** Reductions of attributable premature mortality under the five scenarios were estimated based on the Global Burden of Disease (GBD) study and annual PM_2.5_ values at a resolution of 0.625º × 0.5º. Data from the Hong Kong, Macao, and Taiwan are not available in this study.


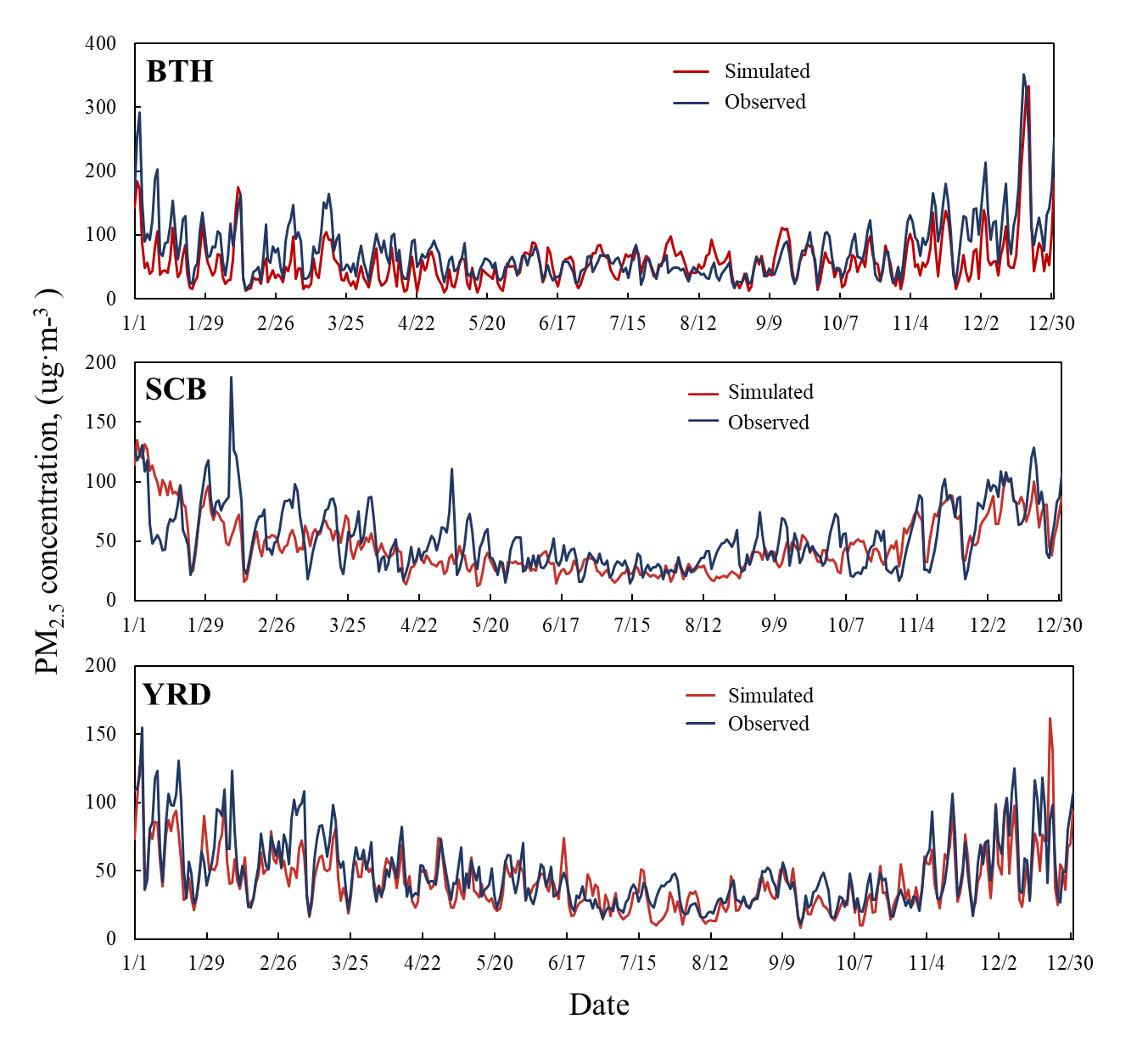


**Fig. S9. Evaluation of GEOS-Chem PM_2.5_ simulation.** Time series of daily mean observed and simulated PM_2.5_ concentrations for the three megacity clusters, BTH, SCB, and YRD, producing correlation coefficients of 0.81, 0.68, and 0.83, respectively.


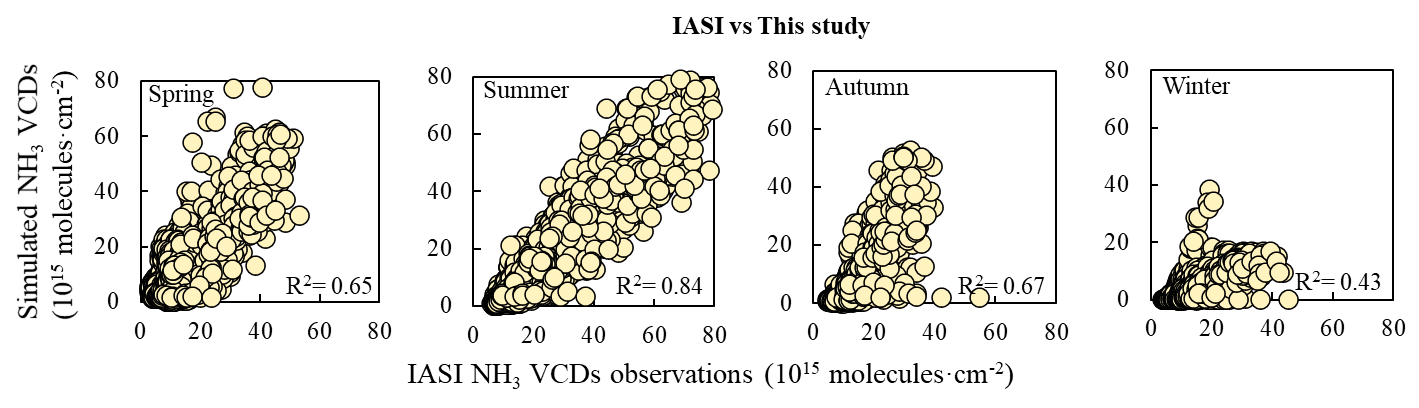


Fig. S10. Correlations between the IASI NH_3_ VCDs and those simulated by GEOS-Chem at a resolution of 0.625º×0.5º for spring, summer, autumn, and winter, respectively.

**Table S1. Some key documents on large-scale farming issued by the Chinese Government in the past decade**

| Time | Policy | Website (accessed by May 15,2024) |
| --- | --- | --- |
| 2014 | *Opinions on Guiding the Orderly Transfer of Rural Land Management Rights and Developing Moderate Scale Agricultural Operations* | https://www.gov.cn/gongbao/content/2014/content_2786719.htm |
| 2015 | *Guiding Opinions on Promoting Moderate Scale Agricultural Operations through Comprehensive Agricultural Development* | https://www.gov.cn/zhengce/2016-05/25/content_5076559.htm |
| 2015 | *Opinions on Supporting Various Forms of Moderate Scale Operations to Promote the Transformation of Agricultural Development Methods* | https://www.mof.gov.cn/gp/xxgkml/nys/201508/t20150803_2512247.htm |
| 2016 | *Opinions on Improving the Methods for Separating Ownership Rights, Contractual Rights, and Management Rights of Rural Land* | https://www.gov.cn/gongbao/content/2016/content_5133019.htm |
| 2016 | *No. 1 Central Document: Laying down Several Opinions on Carrying out New Concept of Development and Speeding up Agricultural Modernization to Realize the Goal of Moderately Prosperous Society* | https://www.gov.cn/gongbao/content/2016/content_5045927.htm |
| 2016 | *National Agricultural Modernization Plan (2016-2020)* | https://www.gov.cn/zhengce/zhengceku/2016-10/20/content_5122217.htm |
| 2016 | *The Outline of the 13th five-year plan for economic and social development of China* | https://www.gov.cn/xinwen/2016-03/17/content_5054992.htm |
| 2017 | *Report of the 19th National Congress of the Communist Party of China* | https://www.gov.cn/zhuanti/2017-10/27/content_5234876.htm |
| 2017 | *No. 1 Central Document: Laying down Several Opinions on Deepening Supply-side Structural Reform in Agriculture and Accelerating the Cultivation of New Growth Engines in Agriculture and Rural Areas* | https://www.gov.cn/zhengce/2017-02/05/content_5165626.htm |
| 2017 | *National Land Consolidation Plan (2016-2020)* | https://www.ndrc.gov.cn/fggz/fzzlgh/gjjzxgh/201705/t20170517_1196769.html |
| 2018 | *No. 1 Central Document: Laying down Opinions of the Central Committee of Communist Party of China and the State Council on Implementing the Strategy of Rural Revitalization* | https://www.gov.cn/zhengce/2018-02/04/content_5263807.htm |
| 2019 | *No. 1 Central Document: Laying down the Opinions of the Central Committee of the Communist Party and the State Council of China on Prioritizing Development of Agriculture and Rural Areas and Addressing the Issues Relating to Agriculture, Rural Areas and Rural People.* | https://www.gov.cn/zhengce/2019-02/19/content_5366917.htm |
| 2019 | *Opinions on Promoting the Organic Connection between Small Farmers and Modern Agriculture Development* | https://www.gov.cn/zhengce/2019-02/21/content_5367487.htm |
| 2020 | *No. 1 Central Document: laying down Opinions of the Central Committee of Communist Party and the State Council of China on Doing a Good Job in the Key Areas of Agriculture, Rural Areas, and Farmers to Ensure the Building of a Moderately Prosperous Society in All Respects.* | https://www.gov.cn/gongbao/content/2020/content_5480477.htm |
| 2020 | *National Plan for Rural Industrial Development (2020-2025)* | https://www.gov.cn/zhengce/zhengceku/2020-07/17/content_5527720.htm |
| 2021 | *No. 1 Central Document: Laying down Opinions of the Central Committee of Communist Party and the State Council of China on Comprehensively Promoting Rural Revitalization and Accelerating the Modernization of Agriculture and Rural Areas* | https://www.gov.cn/zhengce/2021-02/21/content_5588098.htm |
| 2021 | *The Outline of the 14th five-year plan for economic and social development and longrange objectives through the year 2035 of China* | https://www.gov.cn/xinwen/2021-03/13/content_5592681.htm |
| 2021 | *National Overall Plan for the Construction of High-standard Farmland (2021-2030)* | https://www.ndrc.gov.cn/fggz/fzzlgh/gjjzxgh/202111/t20211102_1302810.html |
| 2021 | *14th Five-Year National Animal Husbandry and Veterinary Industry Development Plan* | https://www.gov.cn/xinwen/2021-12/22/content_5663946.htm |
| 2021 | *Administrative Measures for the Circulation of Rural Land Management Rights* | https://www.gov.cn/gongbao/content/2021/content_5600084.htm |
| 2022 | *14th Five-Year-Plan to Advance Agricultural and Rural Modernization* | https://www.gov.cn/zhengce/content/2022-02/11/content_5673082.htm |
| 2022 | *No. 1 Central Document: Laying down Opinions of the Central Committee of Communist Party and the State Council of China on Doing a Good Job in the Key Work of Comprehensively Promoting Rural Revitalization in 2022.* | https://www.gov.cn/zhengce/2022-02/22/content_5675035.htm |
| 2022 | *Report of the 20th National Congress of the Communist Party of China (CPC)* | https://www.gov.cn/xinwen/2022-10/25/content_5721685.htm |
| 2023 | *No. 1 Central Document: Laying down Opinions of the Central Committee of Communist Party and the State Council of China on Comprehensively Promoting Key Works of Rural Revitalization in 2023.* | https://www.gov.cn/xinwen/2023-02/13/content_5741370.htm |
| 2023 | *National Modern Facility Agriculture Construction Plan (2023-2030)* | https://www.gov.cn/zhengce/zhengceku/202306/content_6887551.htm |

Table S2. Threshold numbers defining the category of large-scale livestock farms

| Category | Number |
| --- | --- |
| Beef cattle | 100 (slaughtered) |
| Dairy cattle | 100 (stock) |
| Pigs | 500 (slaughtered) |
| Sheep/goats | 500 (slaughtered) |
| Laying hens | 10000 (stock) |
| Broiler chickens | 50000 (slaughtered) |

Table S3. Specific emissions (Tg) of NH_3_ sources in China

| Category | Subcategory | Emissions (Tg) |
| --- | --- | --- |
| Fertilizer application  - Fertilizer type | Urea | 2.1 |
|  | Ammonium bicarbonate | 0.7 |
|  | Diammonium phosphate | 0.2 |
|  | NPK compound fertilizer | 1.3 |
|  | Other | 0.6 |
| Fertilizer application  - Crop type | Maize | 1.4 |
|  | Wheat | 0.9 |
|  | Rice | 0.8 |
|  | Vegetables | 0.9 |
|  | Fruits | 0.4 |
|  | Others | 0.6 |
| Livestock waste  - Livestock type | Fattening pig and sow | 0.9 |
|  | Beef and dairy cows | 2.1 |
|  | Goat and sheep | 1.3 |
|  | Poultry | 1.2 |
|  | Horse/donkey/mule | 0.3 |
|  | Other | 0.1 |
| Livestock waste  - Breeding system | Free | 3.8 |
|  | Large-scale farming | 1.4 |
|  | Grazing | 0.7 |
| Others | Traffic | 0.1 |
|  | Nitrogen fertilizer production | 0.2 |
|  | Res./ Com. Biomass burning | 0.3 |
|  | Synthetic ammonia | 0.4 |
|  | Res./ Com. Coal combustion | 0.3 |
|  | Agriculture soil | 0.3 |
|  | Others | 0.4 |

Table S4. PM_2.5_ reductions under different NH_3_ mitigation scenarios in China

|  | TECH | RFLW | NM | NMLF–PILOT | NMLF–ALL |
| --- | --- | --- | --- | --- | --- |
| **Reductions of PM_2.5_ concentration (%)** | | | | | |
| China | 6.2 | 4.2 | 10.0 | 19.2 | 22.8 |
| BTH | 6.0 | 3.4 | 9.4 | 23.3 | 24.3 |
| SCB | 5.8 | 4.4 | 9.8 | 20.5 | 24.7 |
| YRD | 6.7 | 4.1 | 10.4 | 22.9 | 25.4 |
| PILOT | 6.2 | 4.0 | 10.0 | 23.7 | 25.3 |
| **Reductions of PM_2.5_ concentration (μg·m^-3^)** | | | | | |
| China | 1.00 | 0.67 | 1.61 | 3.10 | 3.7 |
| BTH | 3.44 | 1.97 | 5.39 | 13.39 | 14.0 |
| SCB | 2.69 | 2.07 | 4.58 | 9.59 | 11.6 |
| YRD | 2.87 | 1.74 | 4.43 | 9.77 | 10.8 |
| PILOT | 2.45 | 1.57 | 3.94 | 9.40 | 10.0 |

Table S5. PM_2.5_-attributable premature deaths avoided by implementing different NH_3_ abatement scenarios

| Scenarios | Avoided PM_2.5_-attributable premature deaths (thousand persons) | | | | |
| --- | --- | --- | --- | --- | --- |
|  | COPD | IHD | LC | Stroke | Sum |
| TECH | 9.6  (8.39-11.62) | 27.06  (23.67-30.57) | 7.41  (6.43-8.48) | 30.22  (26.16-34.58) | 74.31  (64.65-85.25) |
| RFLW | 6.33  (5.51-7.63) | 17.72  (15.51-20.02) | 4.86  (4.22-5.57) | 19.72  (17.07-22.57) | 48.64  (42.31-55.80) |
| NM | 15.62  (13.60-18.85) | 43.87  (38.38-49.57) | 12.01  (10.43-13.75) | 48.89  (42.32-55.95) | 120.40  (104.74-138.12) |
| NMLF–PILOT | 32.85  (28.61-39.64) | 92.82  (81.21-104.86) | 25.33  (22.00-29.00) | 105.17  (91.05-120.35) | 256.18  (222.87-293.85) |
| NMLF–ALL | 39.04  (33.99-47.10) | 109.91  (96.16-124.18) | 30.05  (26.10-34.41) | 123.01  (106.49-140.77) | 302.02  (262.74-346.46) |

Note: COPD, IHD, and IC represent chronic obstructive pulmonary disease, ischemic heart disease, and lung cancer, respectively.

Table S6. Cost–benefits of different NH_3_ mitigation scenarios using low-level economic parameters (USD billions)

|  | | **TECH** | **RFLW** | **NM** | **NMLF**–**PILOT** | **NMLF**–**ALL** |
| --- | --- | --- | --- | --- | --- | --- |
| **Costs** | **Total costs** | **9.3** | **/** | **8.0** | **19.0** | **30.1** |
|  | Using enhanced-efficiency fertilizers | 2.2 | / | 2.1 | 2.1 | 1.9 |
|  | Deep fertilizer placement | 2.2 | / | 2.1 | 0.5 | 0.6 |
|  | Manure management | 5.0 | / | 3.8 | 2.8 | 2.4 |
|  | Large-scale crop farming | / | / | / | 7.7 | 13.2 |
|  | Large-scale livestock farming | / | / | / | 5.8 | 11.9 |
| **Benefits** | **Total benefits** | **20.0** | **10.7** | **29.6** | **84.6** | **117.7** |
|  | **Total private benefits** | **5.4** | **/** | **4.7** | **31.2** | **54.1** |
|  | Reducing overuse of N fertilizer | 1.1 | / | 1.1 | 4.8 | 6.6 |
|  | Deep fertilizer placement | 1.6 | / | 1.5 | 2.0 | 2.4 |
|  | Low crude protein feeding | 0.3 | / | 0.3 | 0.3 | 0.4 |
|  | Manure management | 2.4 | / | 1.8 | 2.9 | 3.9 |
|  | Benefits from large-scale crop farming except for reduction of chemical fertilizer | / | / | / | 7.9 | 15.0 |
|  | Profits expansion after large-scale livestock farming | / | / | / | 13.3 | 25.7 |
|  | **Total societal benefits** | **14.5** | **10.7** | **25.0** | **53.4** | **63.7** |
|  | GHG mitigation benefit | 0.4 | 1.4 | 1.7 | 2.3 | 3.5 |
|  | Human health benefit | 16.2 | 10.6 | 26.2 | 55.7 | 65.7 |
|  | Others | -2.0 | -1.2 | -2.9 | -4.6 | -5.5 |

Table S7. Cost–benefits of different NH_3_ mitigation scenarios using high-level economic parameters (USD billions)

|  | | **TECH** | **RFLW** | **NM** | **NMLF**–**PILOT** | **NMLF**–**ALL** |
| --- | --- | --- | --- | --- | --- | --- |
| **Costs** | **Total costs** | **21.2** | **/** | **18.5** | **46.6** | **74.8** |
|  | Using enhanced-efficiency fertilizers | 6.3 | / | 6.1 | 5.9 | 5.3 |
|  | Deep fertilizer placement | 5.1 | / | 4.8 | 0.6 | 0.7 |
|  | Manure management | 9.8 | / | 7.5 | 6.3 | 6.2 |
|  | Large-scale crop farming | / | / | / | 15.4 | 26.4 |
|  | Large-scale livestock farming | / | / | / | 18.4 | 36.2 |
| **Benefits** | **Total benefits** | **39.3** | **19.6** | **55.0** | **128.5** | **176.0** |
|  | **Total private benefits** | **18.1** | **/** | **14.9** | **48.3** | **77.4** |
|  | Reducing overuse of N fertilizer | 1.1 | / | 1.1 | 4.8 | 6.6 |
|  | Deep fertilizer placement | 4.7 | / | 4.5 | 6.0 | 7.1 |
|  | Low crude protein feeding | 4.0 | / | 3.0 | 3.8 | 4.5 |
|  | Manure management | 8.2 | / | 6.3 | 11.4 | 16.4 |
|  | Benefits from large-scale crop farming except for reduction of chemical fertilizer | / | / | / | 9.0 | 17.0 |
|  | Profits expansion after large-scale livestock farming | / | / | / | 13.3 | 25.7 |
|  | **Total societal benefits** | **21.3** | **19.6** | **40.1** | **80.2** | **98.6** |
|  | GHG mitigation benefit | 2.0 | 6.9 | 8.5 | 11.3 | 17.6 |
|  | Human health benefit | 21.3 | 13.9 | 34.5 | 73.5 | 86.6 |
|  | Others | -2.0 | -1.2 | -2.9 | -4.6 | -5.5 |

**Table S8. PM_2.5_ reductions (%) under the NMLF**–**ALL scenario with and without an additional 20% reduction in acidic gas emissions**

| Regions | NMLF–ALL | | NMLF–ALL+20% acidic gas emission reduction | |
| --- | --- | --- | --- | --- |
|  | January | July | January | July |
| China | 17.2 | 30.5 | 19.2 | 36.2 |
| BTH | 8.9 | 40.5 | 11.1 | 45.9 |
| SCB | 19.4 | 25.6 | 22.2 | 32.9 |
| YRD | 17.3 | 32.8 | 18.2 | 38.6 |
| PILOT | 16.9 | 36.1 | 18.8 | 41.9 |

Table S9. Recommended N application rate (kg N·ha^-1^) for vegetables

| Provinces | N application rate | Provinces | N application rate |
| --- | --- | --- | --- |
| Beijing | 270 | Hubei | 225 |
| Tianjin | 300 | Hunan | 225 |
| Hebei | 345 | Guangdong | 210 |
| Shanxi | 300 | Guangxi | 195 |
| Inner Mongolia | 315 | Hainan | 195 |
| Liaoning | 345 | Chongqing | 210 |
| Jilin | 240 | Sichuan | 240 |
| Heilongjiang | 225 | Guizhou | 195 |
| Shanghai | 225 | Yunnan | 195 |
| Jiangsu | 270 | Tibet | 240 |
| Zhejiang | 270 | Shaanxi | 270 |
| Anhui | 270 | Gansu | 270 |
| Fujian | 255 | Qinghai | 270 |
| Jiangxi | 255 | Ningxia | 300 |
| Shandong | 315 | Xinjiang | 330 |
| Henan | 255 |  |  |

Note: Values derived from the guidance on scientific fertilization of major crops in 2010 via the Ministry of Agriculture of the People’s Republic of China, Duan et al. [8] and Zhang et al. [12].

**SI References**

1. Li B, Chen L, Shen W *et al.* Improved gridded ammonia emission inventory in China. *Atmos Chem Phys* 2021;**21**:15883–900.

2. Zhang L, Chen Y, Zhao Y *et al.* Agricultural ammonia emissions in China: reconciling bottom-up and top-down estimates. *Atmos Chem Phys* 2018;**18**:339–55.

3. Kang Y, Liu M, Song Y *et al.* High-resolution ammonia emissions inventories in China from 1980 to 2012. *Atmos Chem Phys* 2016;**16**:2043–58.

4. Huang X, Song Y, Li M *et al.* A high-resolution ammonia emission inventory in China. *Global Biogeochem Cycles* 2012;**26**: GB1030.

5. Kong L, Tang X, Zhu J *et al.* Improved Inversion of Monthly Ammonia Emissions in China Based on the Chinese Ammonia Monitoring Network and Ensemble Kalman Filter. *Environ Sci Technol* 2019;**53**:12529–38.

6. Kurokawa J, Ohara T. Long-term historical trends in air pollutant emissions in Asia: Regional Emission inventory in ASia (REAS) version 3. *Atmos Chem Phys* 2020;**20**:12761–93.

7. Lesiv M, Laso Bayas JC, See L *et al.* Estimating the global distribution of field size using crowdsourcing. *Global Change Biology* 2019;**25**:174–86.

8. Duan J, Ren C, Wang S *et al.* Consolidation of agricultural land can contribute to agricultural sustainability in China. *Nat Food* 2021;**2**:1014–22.

9. A. Jarvis, H. I. Reuter, A. Nelson, E. Guevara, Hole-filled SRTM for the globe Version 4. available from the CGIAR-CSI SRTM 90m Database (http://srtm. csi. cgiar. org) 15, 5 (2008).

10. Wu Y, Xi X, Tang X *et al.* Policy distortions, farm size, and the overuse of agricultural chemicals in China. *Proc Natl Acad Sci USA* 2018;**115**:7010–5.

11. Wang C, Duan J, Ren C *et al.* Ammonia Emissions from Croplands Decrease with Farm Size in China. *Environ Sci Technol* 2022;**56**:9915–23.

12. Zhang X, Gu B, Van Grinsven H *et al.* Societal benefits of halving agricultural ammonia emissions in China far exceed the abatement costs. *Nat Commun* 2020;**11**:4357.

13. Zhang X, Zou T, Lassaletta L *et al.* Quantification of global and national nitrogen budgets for crop production. *Nat Food* 2021;**2**:529–40.

14. Guo Y, Chen Y, Searchinger TD *et al.* Air quality, nitrogen use efficiency and food security in China are improved by cost-effective agricultural nitrogen management. *Nat Food* 2020;**1**:648–58.

15. Ti C, Xia L, Chang SX *et al.* Potential for mitigating global agricultural ammonia emission: A meta-analysis. *Environmental Pollution* 2019;**245**:141–8.

16. Reis S, Howard C, Sutton MA eds. *Costs of Ammonia Abatement and the Climate Co-Benefits*. Dordrecht: Springer Netherlands, 2015.

17. Cao Y, Xing X, Bai Z *et al.* Review on ammonia emission mitigation techniques of crop-livestock production system. *Scientia Agricultura Sinica* 2018;**51**:566–80.

18. Xue L, Liu X, Lu S *et al.* China’s food loss and waste embodies increasing environmental impacts. *Nat Food* 2021;**2**:519–28.

19. Lu S, Cheng G, Li T *et al.* Quantifying supply chain food loss in China with primary data: A large-scale, field-survey based analysis for staple food, vegetables, and fruits. *Resources, Conservation and Recycling* 2022;**177**:106006.

20. National Development and Reform Commission (NDRC). *China Agricultural Machinery Industry Yearbook 2016*. Beijing, China: China Statistics Press, 2017.

21. Chen J. Study on the behavior and influencing factors of resource utilization of manure in pig breeding enterprises in China. *M.D. Thesis.* Chinese Academy of Agricultural Sciences, 2019.

22. Di P. Ecological behavior of pig farmers in Hebei Province Factor study. *M.D. Thesis.* Hebei Agricultural University, 2019.

23. Du Y, Ge Y, Ren Y *et al.* A global strategy to mitigate the environmental impact of China’s ruminant consumption boom. *Nat Commun* 2018;**9**:4133.

24. Gao Y, Liu B, Yu L *et al.* Social capital, land tenure and the adoption of green control techniques by family farms: Evidence from Shandong and Henan Provinces of China. *Land Use Policy* 2019;**89**:104250.

25. Gao Y, Zhang X, Lu J *et al.* Adoption behavior of green control techniques by family farms in China: Evidence from 676 family farms in Huang-huai-hai Plain. *Crop Protection* 2017;**99**:76–84.

26. Hu M. Analysis of factors affecting farmers’ adoption of fertilizer-saving green agricultural technology—Take soil testing and formula fertilization technology as an example. *M.D. Thesis.* Henan Agricultural University, 2021.

27. Huang Z, Chen S, Wang J. An investigation and analysis of pig farmers’ dung14.treatment problems: Based on 284 sample data from four provinces. *Chinese Journal of Animal Science* 2015:72–76.

28. Jiang Y. Study on farmers’ adoption of testing soil for formulated fertilization technology and efficiency — A case of rice producers in Taizhou. *M.D. Thesis.* Nanjing Agricultural University, 2016.

29. Li C, Shi Y, Khan SU *et al.* Research on the impact of agricultural green production on farmers’ technical efficiency: evidence from China. *Environ Sci Pollut Res* 2021;**28**:38535–51.

30. Li P. The influence of the scale of breeding on the high quality development of pig breeding industry. *Ph.D. Thesis.* Chinese Academy of Agricultural Sciences, 2021.

31. Li Y, Fan Z, Jiang G *et al.* Addressing the Differences in Farmers’ Willingness and Behavior Regarding Developing Green Agriculture—A Case Study in Xichuan County, China. *Land* 2021;**10**:316.

32. Liu C, Zhang X, Wang S. Deviation analysis of adoption willingness and application behavior of water-saving irrigation technology in family farm operators. *Journal of Agro-Forestry Economics and Management* 2021;**30**:295–304.

33. Liu C, Zhang X, Zhang W. The adoption behaviors and income effects of soil testing and formula fertilization technology on family farms. *Research of Agricultural Modernization* 2021;**42**:123–31.

34. Liu Y. Research on subsidy policy instrument effect to animal manure treatment methods — Based on the investigation of biogas treatment methods. *M.D. Thesis.* Northwest A & F University, 2018.

35. Liu Y, Ruiz-Menjivar J, Zhang L *et al.* Technical training and rice farmers’ adoption of low-carbon management practices: The case of soil testing and formulated fertilization technologies in Hubei, China. *Journal of Cleaner Production* 2019;**226**:454–62.

36. Liu Z. Study on resource utilization behavior and influencing factors of pig manure in Hebei province. *M.D. Thesis.* Hebei Agricultural University, 2021.

37. Niu Z, Chen C, Gao Y *et al.* Peer effects, attention allocation and farmers’ adoption of cleaner production technology: Taking green control techniques as an example. *Journal of Cleaner Production* 2022;**339**:130700.

38. Pan D, Tang J, Zhang L *et al.* The impact of farm scale and technology characteristics on the adoption of sustainable manure management technologies: Evidence from hog production in China. *Journal of Cleaner Production* 2021;**280**:124340.

39. Qi X, Liang F, Yuan W *et al.* Factors influencing farmers’ adoption of eco-friendly fertilization technology in grain production: An integrated spatial–econometric analysis in China. *Journal of Cleaner Production* 2021;**310**:127536.

40. Tong D, Huang W, Ying R. The impacts of grassroots public agricultural technology extension onfarmers’technology adoption: An empirical analysis of rice technology demonstration. *China Rural Survey* 2018:59–73.

41. Wang W, Zhang Y. Farmers’ willingness and behavior of replacing chemical fertilizer with organic fertilizer from the perspective of scale differentiation ——based on the survey data of citrus farmers in the main production areas of Sichuan and Chongqing. *Chinese Journal of Agricultural Resources and Regional Planning* 2022;**43**:46–57.

42. Xia W, Du Z, Gao L. Study on the impact of land operational scale on the application of formula fertilization technology by soil testing: based on the observation from family farm monitoring data. *China Land Science* 2019;**33**:70–8.

43. Xue W, Li X, Huo X. Can agricultural technology training promote the adoption of soil testing and formula fertilization technology for apple growers?—From the perspective of different land size. *World Agriculture* 2021:49–57.

44. Yan Y, Chen M, Zhang J *et al.* Farmers’ willingness and behavior response to environmental friendly cultivated land protection technology: the empirical evidence from application of soil testing and formula fertilization technology based on 1092 farmers in Jiangxi province. *China Land Science* 2021;**35**:85–93.

45. Yu C. Study on cleaner production behavior of large-scale pig farms. *Ph.D. Thesis.* Shandong Agricultural University*,* 2019.

46. Yu L, Chen C, Niu Z *et al.* Risk aversion, cooperative membership and the adoption of green control techniques: Evidence from China. *Journal of Cleaner Production* 2021;**279**:123288.

47. Yu L, Zhao D, Xue Z *et al.* Research on the use of digital finance and the adoption of green control techniques by family farms in China. *Technology in Society* 2020;**62**:101323.

48. Yue J, Cai Y, Wu J. On the impact of contract farming on family farms’ adoption of environmentally friendly technologies. *Journal of Zhejiang A&F University* 2022;**39**:207–13.

49. Zeng Y. Environmental regulation, social norm and scale breeding farmers’ cleaner production behaviors. *Ph.D. Thesis.* Huazhong Agricultural University*,* 2020.

50. Zhang Z. The effects of environmental regulation on livestock manure utilization behavior of farmers. *M.D. Thesis.* Northwest A & F University, 2021.

51. Cai Y, Du Z. Ecological Consciousness of Family Farms’ Production Behavior and Analysis of Its Influencing Factors. *Chinese Rural Economy* 2016:33–45.

52. Naylor R, Steinfeld H, Falcon W *et al.* Losing the Links Between Livestock and Land. *Science* 2005;**310**:1621–2.

53. Zhang Q, Zheng Y, Tong D *et al.* Drivers of improved PM_2.5_ air quality in China from 2013 to 2017. *Proc Natl Acad Sci USA* 2019;**116**:24463–9.

54. Zhang Q, Jiang X, Tong D *et al.* Transboundary health impacts of transported global air pollution and international trade. *Nature* 2017;**543**:705–9.

55. Ma R, Li K, Guo Y *et al.* Mitigation potential of global ammonia emissions and related health impacts in the trade network. *Nat Commun* 2021;**12**:6308.

56. Zhai S, Jacob DJ, Wang X *et al.* Control of particulate nitrate air pollution in China. *Nat Geosci* 2021;**14**:389–95.

57. Dang R, Liao H. Severe winter haze days in the Beijing–Tianjin–Hebei region from 1985 to 2017 and the roles of anthropogenic emissions and meteorology. *Atmos Chem Phys* 2019;**19**:10801–16.

58. Yin H, Brauer M, Zhang J (Jim) *et al.* Population ageing and deaths attributable to ambient PM_2.5_ pollution: a global analysis of economic cost. *The Lancet Planetary Health* 2021;**5**:e356–67.

59. Burnett R, Chen H, Szyszkowicz M *et al.* Global estimates of mortality associated with long-term exposure to outdoor fine particulate matter. *Proc Natl Acad Sci USA* 2018;**115**:9592–7.

60. Geng G, Zheng Y, Zhang Q *et al.* Drivers of PM_2.5_ air pollution deaths in China 2002–2017. *Nat Geosci* 2021;**14**:645–50.

61. Liu M, Huang X, Song Y *et al.* Ammonia emission control in China would mitigate haze pollution and nitrogen deposition, but worsen acid rain. *Proc Natl Acad Sci USA* 2019;**116**:7760–5.

62. National Development and Reform Commission (NDRC), China Agricultural Products Cost-Beneﬁt Yearbook 2016 (China Statistics Press: Beijing, China, 2017).

63. Xie Y, Dai H, Zhang Y *et al.* Comparison of health and economic impacts of PM_2.5_ and ozone pollution in China. *Environment International* 2019;**130**:104881.

64. Sutton MA, Howard C, Erisman JW. *The European Nitrogen Assessment: Sources, Effects and Policy Perspectives.* Cambridge University Press, 2011.

65. Zhang W, Dou Z, He P *et al.* New technologies reduce greenhouse gas emissions from nitrogenous fertilizer in China. *Proc Natl Acad Sci USA* 2013;**110**:8375–80.

66. West JJ, Smith SJ, Silva RA *et al.* Co-benefits of mitigating global greenhouse gas emissions for future air quality and human health. *Nature Clim Change* 2013;**3**:885–9.

67. Wang Y, Zhou J, Wang G *et al.* Advances in low-protein diets for swine. *J Animal Sci Biotechnol* 2018;**9**:60.

68. Wang Q, Awasthi MK, Ren X *et al.* Comparison of biochar, zeolite and their mixture amendment for aiding organic matter transformation and nitrogen conservation during pig manure composting. *Bioresource Technology* 2017;**245**:300–8.

69. Wang R, Lu S, Zhou L *et al.* Assessing nutritional and economic aspects of food loss and waste in China. *Sustainable Production and Consumption* 2023;**42**:95–105.

70. Cattaneo A, Sánchez MV, Torero M *et al.* Reducing food loss and waste: Five challenges for policy and research. *Food Policy* 2021;**98**:101974.
